# Activating Inducible T-cell Costimulator Yields Antitumor Activity Alone and in Combination with Anti-PD-1 Checkpoint Blockade

**DOI:** 10.1158/2767-9764.CRC-22-0293

**Published:** 2023-08-16

**Authors:** Sapna Yadavilli, Jeremy D. Waight, Sara Brett, Meixia Bi, Tianqian Zhang, Yao-Bin Liu, Catherine Ellis, David C. Turner, Ashleigh Hahn, Hong Shi, Laura Seestaller-Wehr, Junping Jing, Qing Xie, Jafar Sadik Shaik, Xiao Ji, Robert Gagnon, William Fieles, Laura Hook, Steven Grant, Stephanie Hopley, M. Phillip DeYoung, Christina Blackwell, Michael Chisamore, Robert Biddlecombe, David J. Figueroa, Christopher B. Hopson, Roopa Srinivasan, James Smothers, Michele Maio, Danny Rischin, Daniel Olive, Elaine Paul, Patrick A. Mayes, Axel Hoos, Marc Ballas

**Affiliations:** 1 GSK, Collegeville, Pennsylvania.; 2 GSK, Stevenage, Hertfordshire, United Kingdom.; 3 Merck & Co., Inc., Rahway, New Jersey.; 4 University of Siena and Center for Immuno-Oncology, Azienda Ospedaliera Universitaria Senese, Siena, Italy.; 5 Department of Medical Oncology, Peter MacCallum Cancer Centre, Melbourne, Victoria, Australia.; 6 Sir Peter MacCallum Department of Oncology, University of Melbourne, Melbourne, Victoria, Australia.; 7 CRCM, Immunity and Cancer, Inserm, U1068, Institut Paoli-Calmettes, Aix-Marseille Université, UM105, CNRS, UMR7258, Marseille, France.

## Abstract

In recent years, there has been considerable interest in mAb-based induction of costimulatory receptor signaling as an approach to combat cancer. However, promising nonclinical data have yet to translate to a meaningful clinical benefit. Inducible T-cell costimulator (ICOS) is a costimulatory receptor important for immune responses. Using a novel clinical-stage anti-ICOS immunoglobulin G4 mAb (feladilimab), which induces but does not deplete ICOS+ T cells and their rodent analogs, we provide an end-to-end evaluation of the antitumor potential of antibody-mediated ICOS costimulation alone and in combination with programmed cell death protein 1 (PD-1) blockade. We demonstrate, consistently, that ICOS is expressed in a range of cancers, and its induction can stimulate growth of antitumor reactive T cells. Furthermore, feladilimab, alone and with a PD-1 inhibitor, induced antitumor activity in mouse and humanized tumor models. In addition to nonclinical evaluation, we present three patient case studies from a first-time-in-human, phase I, open-label, dose-escalation and dose-expansion clinical trial (INDUCE-1; ClinicalTrials.gov: NCT02723955), evaluating feladilimab alone and in combination with pembrolizumab in patients with advanced solid tumors. Preliminary data showing clinical benefit in patients with cancer treated with feladilimab alone or in combination with pembrolizumab was reported previously; with example cases described here. Additional work is needed to further validate the translation to the clinic, which includes identifying select patient populations that will benefit from this therapeutic approach, and randomized data with survival endpoints to illustrate its potential, similar to that shown with CTLA-4 and PD-1 blocking antibodies.

**Significance:**

Stimulation of the T-cell activation marker ICOS with the anti-ICOS agonist mAb feladilimab, alone and in combination with PD-1 inhibition, induces antitumor activity across nonclinical models as well as select patients with advanced solid tumors.

## Introduction

Tumors employ a range of mechanisms to evade immune-mediated clearance (1–3). As a prime example, immune checkpoint pathways are often co-opted by tumors to impair effective antitumor immune responses (1, 2). With the advent of immunotherapies designed to block inhibitory receptors such as CTL-associated protein-4 (CTLA-4 or CD152) and programmed cell death protein 1 (PD-1 or CD279), a significant amount of progress has been made in the realm of immune evasion (1, 2, 4). Despite the success of checkpoint-based immunotherapy in a range of indications, many patients show innate or acquired therapeutic resistance (1, 4), underscoring the multifaceted nature by which tumors evade immune-mediated destruction. Indeed, the cancer immunity cycle posits that multiple factors, including both coinhibitory and costimulatory receptors, are required for effective antitumor immune responses (5, 6). Thus, complementary approaches beyond blockade of immune checkpoints, such as triggering costimulatory receptors, may have therapeutic potential.

By virtue of a range of nonclinical evidence, costimulatory receptors have garnered significant interest as therapeutic targets, each harboring considerations for therapeutic utility (2, 5). Among others, inducible T-cell costimulator (ICOS or CD278) is a costimulatory receptor belonging to the CD28 immunoglobulin (Ig) receptor superfamily, which includes CTLA-4 and PD-1 (7). Unlike CD28, ICOS expression is low on naïve T cells but is upregulated upon T-cell receptor (TCR) stimulation (7, 8). ICOS signaling induces the production of type 1 and 2 T helper (Th1 and Th2) cytokines, and has a pivotal role in T-cell proliferation, differentiation, survival, and function during antigen-stimulated immune responses (7, 9–12). ICOS is expressed on a subset of CD4^+^ T cells, CD8^+^ cytotoxic T cells, and most regulatory T (T_reg_) cells in tumor-infiltrating lymphocytes (TIL), across several indications. Accordingly, previous nonclinical research supports the concept that costimulation of T cells using recombinant ICOS ligand (ICOS-L or CD275) or agonist mAbs has significant antitumor activity (13, 14).

Feladilimab, a novel, humanized, clinical-stage anti-ICOS IgG4 mAb, is the first ICOS agonist mAb to be tested in first-in-human clinical trials; it was chosen for its selective binding to the ICOS receptor, which leads to stimulation of the ICOS signaling pathway and subsequent T-cell activation. This particular IgG4 isotype contains two amino acid substitutions (IgG4-PE; refs. 15, 16), which minimize Fab-arm exchange for hinge stabilization and attenuates binding affinity of the fragment crystallizable (Fc) region to both activating Fcγ receptors and complement component 1q (2, 17). Despite these modifications, feladilimab retains binding to the inhibitory Fcγ receptor, FcγRIIb (CD32B), which has been shown to be an important feature for optimal function of several agonist antibodies (18, 19). Consistent with CTLA-4 and PD-1 blockade, the effects of feladilimab on T-cell activation are anticipated to modulate T-cell dynamics, promote immune-mediated tumor regression, and improve survival of patients whose tumors have intrinsically activated T cells or whose immune systems are primed, either by prior treatments or rational combination of therapies that promote immune priming, such as anti-CTLA-4 or anti-PD-1 mAbs, chemotherapy, or radiotherapy (2, 20–23). Because tumor-reactive CD4^+^ T cells coexpress ICOS and PD-1 (24, 25), combining feladilimab with anti-PD-1 mAbs has the potential for greater antitumor activity than either agent alone.

Here, we discuss the expression and prognostic value of ICOS in solid tumors, outline the functional effects of ICOS agonism, and establish the unique mechanistic profile of feladilimab and its antitumor potential alone and in combination with PD-1 blockade in nonclinical models. We also report three patient case studies from an open-label, phase I clinical trial (INDUCE-1; NCT02723955; primary analyses not yet conducted). This study is designed to evaluate the safety and tolerability of feladilimab as monotherapy and in combination with the PD-1 inhibitor pembrolizumab in patients with advanced solid tumors; with an additional aim to identify the recommended phase II dose. Together, these data provide a spectrum of investigation for targeting ICOS (from nonclinical to clinical), supporting the therapeutic concept of antibody-based ICOS co-stimulation. However, more widespread clinical investigations are needed to optimize the selection of patient populations as well as the optimal dose and schedule, which are a challenge for agonist antibodies for ICOS-based therapeutic approaches (2).

## Materials and Methods

### Cell Lines and Primary Cell Cultures

Murine mammary carcinoma EMT6 [CRL-2755, Research Resource Identifier (RRID): CVCL_1923], human melanoma A2058 (CRL-11147, RRID:CVCL_1059), and non–small cell lung carcinoma (NSCLC) A549 (CCL-185, RRID:CVCL_0023) tumor lines were acquired from ATCC. The wild-type myeloma Ba/F3 tumor line was obtained from the DSMZ (ACC-300, RRID:CVCL_0161). All cell lines were expanded upon receipt and batch frozen at a low passage number (≤10) for *in vivo* studies. Prior to *in vivo* use, cell lines were tested for a panel of pathogens including *Mycoplasma* (Charles Rivers Laboratory, Infectious Disease PCR Mouse/Rat Comprehensive CLEAR Panel). Cancer patient whole blood and surgically resected tumor tissue was obtained from Avaden Biosciences. For healthy donor human peripheral blood mononuclear cell (PBMC) analysis, whole blood was derived from volunteers within the GSK on-site blood donation unit. All donor blood was acquired with appropriate consent and in accordance with GSK Human Biological Sample Management policies. In each case (i.e., healthy donor or cancer patient), whole blood was collected in sodium heparin tubes (BD Biosciences or Sagent Pharmaceuticals). PBMCs were isolated from whole blood by density gradient centrifugation through Histopaque (Sigma-Aldrich) or Sepmate^TM^ (Stemcell Technologies). Where needed, further separation of T-cell subsets was conducted by negative selection using magnetic bead–based isolation (Dynabeads^TM^ Life Technologies or RosetteSep/EasySep, Stemcell Technologies). Fresh PBMC for NSG^TM^ mice engrafting were purchased from AllCells.

### Antibodies

The following antihuman antibodies were used for flow cytometry analysis: CD4 (RRID:AB_1645271), CD8 (RRID:AB_893422), CD69 (RRID:AB_314846), Ki67 (RRID:AB_396302), ICOS (RRID:AB_10732348), FOXP3 (RRID:AB_1518782), PD-1 (RRID:AB_940475), CD25 (RRID:AB_11218989), CD45 (RRID:AB_1645452), and CD3 (RRID:AB_493741). The following anti-mouse antibodies were used for flow cytometry analysis: CD3 (RRID:AB_394595), CD4 (RRID:AB_393977), CD8 (RRID:AB_1645237), CD25 (RRID:AB_394509), FOXP3 (RRID:AB_469457), ICOS (RRID:AB_2562545), Ki67 (RRID:AB_2564285), PD-1 (RRID:AB_57201), T-bet (RRID:AB_10564071), granzyme B (RRID:AB_2294995), CD127 (RRID:AB_1937216), CD45 (RRID:AB_2737976), and CD44 (RRID:AB_10895752). All human and murine flow cytometry studies incorporated fixable Near IR Live/Dead (Invitrogen) dye to assess viability.

For Western-based characterization of protein kinase B (AKT) signaling, antibodies against pAKT (RRID:AB_2315049), total AKT (RRID:AB_329827), pGSK3β (RRID:AB_10013750), total GSK3β (RRID:AB_2636978), and ICOS (discontinued, no RRID) were used. The following antibodies were used for immunofluorescence of case study tumor samples: ICOS (RRID:AB_10710236), PD-1 (RRID:AB_2894867), PD-L1 (RRID:AB_2827816), OX40/CD134 (RRID:AB_10639951), CD16 (RRID:AB_2104009), CD56 (RRID:AB_2864402), Ki67 (RRID:AB_1661312), HLA-DR (RRID:AB_964563), granzyme B (RRID:AB_2114697), CD8 (RRID:AB_2075537), CD3 (RRID:AB_2631163), CD4 (RRID:AB_2750883), S100 (RRID:AB_2811056), and PanCytokeratin (RRID:AB_2134432). All antibody clones and manufacturers for *in vitro* assays are listed in Supplementary Table S1.

The following anti-murine antibodies were used for *in vivo* tumor studies: ICOS (7E.17G9 mIgG1, kappa, Absolute Antibody Ltd; 7E.17G9 rIgG2b, Bioxcell, RRID:AB_1107622) and PD-1 (RMP1-14 rIgG2a, BioXcell, RRID:AB_10949053). 7E.17G9 mIgG1 is a recombinant mAb to ICOS that was generated using Absolute Antibody's Recombinant Platform with variable regions (i.e., specificity) from the hybridoma 7E.17G9 (raised by immunizing rats); the chimeric antibody contains rat-derived variable antibody regions and mouse-derived constant regions.

### Flow Cytometry

In all flow cytometry experiments using primary cells, nonspecific binding was blocked with human or mouse Fc block (Miltenyi Biotec) on ice for 30 minutes prior to incubation with detection antibodies. Intracellular staining was conducted using the Transcription Factor Buffer set (BD Biosciences), per manufacturer's instructions. Following fluorescence compensation, data were acquired on FACS Canto II or Fortessa (BD Biosciences) and analyzed with FACSDiva (BD Biosciences, RRID:SCR_001456) or Flowjo (BD Biosciences, RRID:SCR_008520) software.

### The Cancer Genome Atlas and Single-cell RNA Sequencing Analysis

Public gene expression data from The Cancer Genome Atlas (TCGA) was used to evaluate *ICOS*, ICOS-L (*ICOSLG*), and PD-L1 (*CD274*) expression in approximately 16 tumor indications. Data were retrieved from TCGA (B37 assembly), using an internal copy from OmicSoft (accessed May 1, 2018). Heat maps of normalized FPKM (fragments per kilobase of transcript per million mapped reads) expression data were generated using ArrayStudio (OmicSoft). Tumor types (TCGA abbreviations) evaluated were head and neck squamous cell carcinoma (HNSC), lung adenocarcinoma (LUAD), lung squamous cell carcinoma (SC, LUSC), esophageal (ESCA), bladder (BLCA), pancreatic (PAAD), breast (BRCA), cervical (CESC), sarcoma (SARC), and melanoma (SKCM; TCGA, RRID:SCR_003193).

ICOS expression on individual cell subsets was evaluated using publicly available RNA sequencing (RNA-seq) datasets from melanoma, head and neck squamous cell carcinoma (HNSCC), and NSCLC (GSE72056, GSE103322, and GSE99254, respectively; refs. 26–28). Data were analyzed as described and reported as log_2_ transcript count per million (TPM).

Correlative analysis of ICOS expression and overall survival (OS) rates was extracted from publicly available TCGA and metadata of patients with HNSCC. Data were retrieved from TCGA using an internal copy from OmicSoft (accessed January 23, 2020). Data were from a heterogenous patient population including patients with stage I–IV disease. Groups were determined by top (ICOS^high^) versus bottom (ICOS^low^) quartile [log_2_(FPKM+0.1)]; RNA expression was generated from samples derived from primary tumors only. For some analyses, metadata allowed for dissection of patients based on human papillomavirus (HPV) status. Patients with indeterminate HPV status were excluded.

### Generation of Feladilimab, and Surface Plasmon Resonance and Biolayer Interferometry

Feladilimab is a humanized variant of the murine mAb obtained from Institut Paoli-Calmettes, INSERM (Daniel Olive, Marseille, France). The murine antibody was generated by immunization of BALB/c mice with recombinant human ICOS-Fc (COS-7 cell-derived) and subsequent hybridoma production. Following clone screening, the selected clone was humanized and grafted onto an IgG4 hinge-stabilized isotype containing two amino acid substitutions (S228P/L235E), dubbed IgG4-PE (15, 17).

To evaluate feladilimab or recombinant human ICOS-L (His-tagged, Sino Biological) affinity for human ICOS, surface plasmon resonance analysis and biolayer interferometry analyses were performed (details in Supplementary Materials and Methods).

### *In Vitro* Functional Assays

The *in vitro* functional activity of feladilimab was evaluated in a variety of primary human immune cell systems, including PBMC [anti-CD3 (OKT3, eBioscience), anti-CD3/28 (Dynabeads^TM^, Thermo Fisher Scientific), and mixed lymphocyte reaction (MLR)] and dissociated tumor-based assays. To assess feladilimab-mediated enhancement of TCR-stimulated T cells, bulk PBMC derived from healthy donors or patients with cancer (NSCLC, HNSCC, or melanoma) or isolated T-cell subsets (e.g., T_reg_ cells) were polyclonally stimulated with plate-bound anti-CD3 (OKT3, 0.6–1 μg/mL) for 48 hours and exposed to plate-bound feladilimab or cognate isotype control (at doses of 5–12.5 μg/mL) for 24–72 hours at 37°C and 5% CO_2_. Prior to addition of primary cells, antibodies were adhered to round- or flat-bottom 96-well plates (Corning or Falcon) overnight at 4°C or for 4 hours at ambient temperature. Where relevant, cells were also treated alone and in combination with 10–11.1 μg/mL of anti–PD-1 (pembrolizumab, hIgG4) in solution.

Following treatment, cells were characterized for transcriptional changes (NanoString Technologies), activation by cell surface or intracellular flow cytometry, or IFNγ production by Meso Scale Discovery (MSD, Meso Scale Diagnostics, LLC). All analysis of cytokine production (i.e., IFNγ) by MSD was performed on cell-free supernatants. For PBMC from patients with cancer, an overnight rest step was included prior to treatment initiation.

A modified MLR [i.e., allogeneic dendritic cell (DC) MLR] was also used to assess the functional activity of feladilimab alone and in combination with anti-PD-1 (pembrolizumab). Immature monocyte-derived antigen-presenting cells (APC) were first generated by exposing plate-adhered human PBMC to recombinant human GMCSF (1,000 U/mL, PeproTech) and IL4 (500 U/mL, PeproTech) in serum-free AIM-V medium (Thermo Fisher Scientific) for 8 days at 37°C and 5% CO_2_. Isolated peripheral blood CD4^+^ T cells (1 × 10^5^ cells/sample) from an independent human donor were added to the immature APCs at a ratio of 10:1 (T cell:APC) in serum-free AIM-V and stimulated with 0.02 μg/mL of CEFT peptide (JPT) for 48 hours at 37°C and 5% CO_2_. Following prestimulation, test antibodies and associated controls were added in solution (each at 11.1 μg/mL) and incubated for an additional 96 hours at 37°C and 5% CO_2_. Cytokine production in cell-free supernatants was then assessed via MSD.

Primary tumors from patients were dissociated using a gentleMACS^TM^ Dissociator (Miltenyi Biotec). Single-cell suspensions were then expanded in RPMI media (PeproTech) supplemented with human AB serum (Sigma-Aldrich) and recombinant human IL2 (3,000 U/mL, PeproTech) for 15 days as described previously (29). Expanded TILs were incubated with plate-bound anti-CD3 (OKT3, 0.6 μg/mL) and test antibodies [10 μg/mL of plate-bound feladilimab or soluble anti-PD-1 (pembrolizumab)] or controls in round-bottom 96-well plates for 24 hours at 37°C and 5% CO_2_, and fold change in IFNγ production was assessed via MSD.

### Confocal Microscopy

Primary human CD4^+^ T cells (1 × 10^6^ cells/test) were stimulated with anti-CD3/CD28-coated beads (Dynabeads^TM^, at ratios of 1:1, 1:3, 1:5, and 1:10 cells:beads) for 48 hours to induce ICOS expression. For visualization of cellular localization, T cells were then incubated with 3 μg/mL of unlabeled soluble feladilimab or isotype control on ice for 1 hour. The treated cells were then plated in 8-well chamber coverslips and held on ice. Cell cultures were washed in cold media and then transferred to 37°C for various timepoints (0, 15, and 60 minutes) to permit protein trafficking prior to fixation with 4% paraformaldehyde (20 minutes, Sigma). Samples were then pulsed with AlexaFluor 488-conjugated anti-human IgG for 1 hour. To visualize nuclei, ProLong^TM^ Gold mounting media (Invitrogen) containing DAPI was also added to each well. Coverslips were then imaged on a ZEISS LSM 510 Meta Confocal microscope with a 63X oil immersion lens at 1024 × 1024 resolution. Scope settings (i.e., laser power, photomultiplier tube [PMT] gain, offset) were optimized using T = 0 samples. Imaging of all experimental and control samples at all timepoints was done using these settings. For visualization of T cell–DC interactions, T cells were prelabeled on ice for 1 hour with prelabeled (Zenon kit, Invitrogen) Alexa 488 feladilimab, Alexa 568 anti-CD4, and Alexa 647 anti-CD28, and anti-CD11c was used to identify the DCs. Primary antibodies were conjugated to the indicated fluorochromes using manufacturer's instructions (feladilimab, Alexa 488, RRID:AB_2736940; CD4, Alexa 568, RRID:AB_2736951; CD11c, Alexa 532, RRID:AB_2736944; and CD28, Alexa 647, RRID:AB_2736959). DCs (2 × 10^5^/sample) were plated in 8-well chamber coverslips (poly-L-lysine–coated, Sigma-Aldrich) and allowed to attach at room temperature for 1 hour. Coverslips were then placed on ice, the media removed, and the T cells were added at a 1:1 ratio with the adhered DCs and incubated at 37°C for 1 hour and fixed. ProLong Gold mounting media (Invitrogen) was added to each well, then imaging was performed as described above. Methods for Supplementary Movie S1 were as described above, with the exception that T cells were prelabeled with only Alexa 488-labeled feladilimab and images were taken every 5 minutes for 2 hours to obtain time lapse imaging.

### Western Blotting

PBMC-derived human CD4^+^ T cells were activated with CD3/CD28-coated beads (1:20; cell:bead ratio) for 48 hours to induce ICOS expression. CD3/CD28 beads were then removed using EasySep^TM^ magnets (Stemcell Technologies), permitting the cells to rest in the absence of stimulation for 24 hours. Cells were restimulated with 1–10 μg/mL of soluble feladilimab or isotype control for 0.5, 1, 6, and 24 hours at 37°C and then lysed for protein analysis. Similarly, wild type Ba/F3 (murine, DSMZ, ACC-300) or Ba/F3 cells engineered to express human ICOS (termed Ba/F3-huICOS), obtained from Institut Paoli-Calmettes, INSERM (Daniel Olive, Marseille, France), were incubated with 20 μg/mL of soluble feladilimab or isotype control for 1 hour at 37°C. Protein was extracted from primary T cells and Ba/F3 cells using cell lysis buffer (Cell Signaling Technology) supplemented with a 1X protease inhibitor cocktail (Roche). Approximately 25 μg of protein was run on 4%–12% Bis-Tris gels (Invitrogen) and transferred onto nitrocellulose membranes (Invitrogen). Membranes were blocked using LI-COR Odyssey Blocking Buffer and subsequently immunoblotted using the primary and secondary antibodies and scanned on a LI-COR Odyssey imaging system.

### NanoString Analysis

For murine EMT6 pharmacodynamic studies, tumor tissue was harvested from mice 48 hours after second and third dose of indicated mAbs and placed in 50 mL conical tubes containing cold serum-free RPMI1640 (Gibco) and kept on ice until dissociation. Following mechanical dissociation (gentleMACS^TM^ Dissociator), tumor samples were washed with 1X PBS and then lysed by vigorous pipetting in RLT buffer (Qiagen). RNA was extracted from homogenized lysates (QIAshredder, Qiagen) using RNAeasy (Qiagen), per manufacturer's instructions. RNA concentration and quality were then evaluated using Nanodrop (Thermo Fisher Scientific). Approximately 50 ng of total RNA was used for NanoString Mouse PanCancer Immune profiling. Following hybridization overnight at 65°C, the material was transferred to a NanoString cartridge and loaded into the Spring profiler per manufacturer's instructions. Data were then exported and analyzed using nSolver software (NanoString). For heat maps, represented gene cutoff was based on a NanoString signal >20 and a ≥1.5-fold change relative to isotype control.

Similar analyses were conducted on isolated bulk (CD3^+^) T cells (*n* = 6) or CD4^+^ non-T_reg_ or T_reg_ cells (*n* = 4) from healthy human donors. Isolated T cells were incubated in 96-well plates (BD Falcon) precoated with 0.6 μg/mL or 1 μg/mL (non-T_reg_ vs. T_reg_ cells) of mouse anti-human CD3 mAb (OKT3, eBioscience) in the presence or absence of 10 μg/mL feladilimab or isotype control for 24 hours at 37ºC and 5% CO_2_. Following cell stimulation, cells were washed and processed as above for RNA isolation. Approximately 50 ng of total RNA was used for NanoString Human PanCancer Immune profiling.

### Pharmacokinetic Analysis of the mIgG1 anti-ICOS Antibody

For pharmacokinetic analysis of the mIgG1 anti-ICOS antibody (7E.17G9) in EMT6 tumor-bearing mice, serum was collected at 1-hour postdose and both predose and 1-hour postdose at 72 and 144 hours for characterization of antibody presence. Briefly, biotinylated mouse ICOS protein was utilized as the capture reagent and anti-mouse IgG as the detection antibody. Approximately 3 μL of mouse serum was diluted 1/10 with Rexxip A buffer (Gyros Protein Technologies AB) and captured using plate-bound mouse ICOS. The lower limit of quantification was 100 ng/mL, and the higher limit of quantification was 10,000 ng/mL. Quality control samples were stored with study samples and analyzed with each batch of samples against separately prepared calibration standards. At least four of six quality control results and at least 50% of the results from each quality control concentration were required to not deviate from the nominal concentration by more than 25%.

### *Ex Vivo* Human Tumor Explants

Tissue slices (100–300 μm) from patients with HNSCC (*n* = 50) were treated with anti-PD-1 (pembrolizumab, *C*_max_ 65.7 μg/mL) or vehicle control (10 μg/mL) in autologous growth factors for 72 hours and then evaluated for transcriptional changes as described previously (ref. 30; CANscript^TM^, Mitra Biotech/Farcast Biosciences).

### *In Vivo* Tumor Studies

For syngeneic tumor studies, wild type 6–8 weeks old female BALB/c mice (Harlan/Envigo, RRID:IMSR_APB:4790) were implanted subcutaneously with 1 × 10^5^ EMT6 mouse mammary carcinoma cells. Randomization was performed prior to treatment initiation using the Studylog Study Director Suite software, approximately 7–9 days after tumor implantation, when the average tumor volume was approximately 100 mm^3^. ANOVA was used to ensure similarity between groups (*P* > 0.9). Following randomization, EMT6 tumor-bearing mice were administered via intraperitoneal injection biweekly with mouse chimeric or rat anti-mouse ICOS antibody (0.005, 0.05, 0.25, 0.5, 5, 10 mg/kg; 7E.17G9 mIgG1, kappa, Absolute Antibody Ltd and 7E.17G9 rIgG2b, Bioxcell, respectively), anti–PD-1 (10 mg/kg; RMP1-14 rIgG2a, BioXcell) or cognate isotype controls [all from BioXcell; mIgG1 clone MOPC21 (RRID:AB_1107784), rIgG2b clone LTF2 (RRID:AB_1107780), rIgG2a clone 2A3 (RRID:AB_1107769)] for a total of six doses. Doses were converted from μg to mg/kg format for data presentation based on an average mouse weight of 20 g.

For human NSCLC A549 (1 × 10^6^, subcutaneous) and melanoma A2058 (1 × 10^6^, subcutaneous) cell-line derived xenograft studies, 6–8 weeks old female NSG mice (The Jackson Laboratory, RRID:IMSR_JAX:005557) were engrafted intravenously with 20 × 10^6^ human PBMC (single donor; AllCells) when tumors were approximately 100 mm^3^. Approximately 72 hours after engraftment, tumor-bearing mice were administered intraepritoneally with feladilimab (0.04 and 0.4 mg/kg), anti-PD-1 (pembrolizumab, 5 mg/kg), or relevant isotype controls (Eureka therapeutics, ET904) biweekly for a total of six doses. Tumor tissue was harvested, and TILs evaluated by flow cytometry.

Humanized patient xenograft studies were performed in recipient 3-week-old NSG mice, who were engrafted intravenously with CD34^+^ human pluripotent stem cells (HPSC) derived, as described previously, from whole blood from three independent donors (Jackson Laboratory, donors 5003, A*02:01; 5004, A*02:01; 5005, A*01:01) following whole body irradiation, as described previously (31). CD34^+^ HPSC-engrafted mice (*n* = 7–8 mice/group) were implanted with primary human triple-negative breast cancer cells (Jackson Laboratory, BR1126, A*02:01) as described previously. Mice were administered intraperitoneally with feladilimab (0.04 and 0.4 mg/kg) or relevant controls (Eureka therapeutics, ET904) biweekly for the duration of the study.

In all human xenograft studies (NSCLC A549, melanoma A2058, and BR1126 PDX), tumor growth was measured two to three times per week after randomization until only tumor-free mice remained on study. Tumor volume (denoted as mm^3^) was calculated using the formula *length × width^2^ × 0.52*. For the purposes of analysis of survival, in all studies, “death” refers to surpassing a tumor volume cutoff of 2,000 mm^3^. All studies were conducted in accordance with the GSK Policy on the Care, Welfare, and Treatment of Laboratory Animals and were reviewed by the Institutional Animal Care and Use Committee either at GSK or by the ethical review process at the institution where the work was performed.

### IHC and Immunofluorescence

IHC detection of ICOS in tumor samples used 3, 3′-diaminobenzidine for target detection, and sections were counterstained with hematoxylin (BOND polymer refine detection, Leica Biosystems). A multiplexed immunofluorescence (mIF) assay (Clarient MultiOmyx platform; NeoGenomics) was used to evaluate expression of ICOS, PD-1, CD3, CD4, and CD8 among other T-cell markers on formalin-fixed paraffin-embedded tumor tissues obtained from vendors vetted by GSK Human Biological sample group as described above. The iterative process included a round of staining with a Cy3- and Cy5-conjugated antibody (GE Healthcare Life Sciences) and imaging, followed by dye inactivation, background fluorescence imaging, and subtraction of the background before the repeating this cycle for all markers in the panel. For each sample, up to 30 regions of interest were selected under 20x magnification to cover the entire section. Damaged areas (e.g., tissue tears, areas of very high autofluorescence) were avoided. Cell density was reported by phenotype as cells/mm^2^.

### Clinical Study Design

INDUCE-1 (NCT02723955) is an ongoing phase I, multicenter, open-label study that consists of two parts: (Part 1) feladilimab alone and (Part 2) feladilimab in combination with other therapies, including pembrolizumab. Each part includes a dose-escalation (Part 1A and 2A) and dose-expansion phase (Part 1B and 2B). In Part 1A, patients received escalating doses of feladilimab monotherapy (intravenous, day 1, once every 3 weeks) ranging from 0.001 to 10 mg/kg feladilimab. In Part 1B, patients received feladilimab monotherapy (intravenous, day 1, once every 3 weeks) at doses selected for evaluation in the dose-escalation phase and based on their pharmacology. In Part 2A, patients received escalating doses of feladilimab (0.001 to 3 mg/kg) in combination with a fixed dose (200 mg) of pembrolizumab (intravenous, day 1, once every 3 weeks). In Part 2B, patients received feladilimab in combination with a fixed dose (200 mg) of pembrolizumab (intravenous, day 1, once every 3 weeks) at the doses selected for evaluation in the dose-escalation phase and based on their pharmacology.

The study was conducted in accordance with the Declaration of Helsinki and International Conference on Harmonization Good Clinical Practice guidelines. Patients provided written informed consent; all human biological samples were sourced ethically, and their research use was in accordance with the terms of the informed consents under a protocol approved by the relevant Institutional Review Boards/ethics committees.

No significant updates have been made to the protocol with regards to statistical assumptions or methods after trial commencement.

### Clinical Pharmacodynamic Assessments

For pharmacodynamic analyses, tumor tissue was collected predose and at week 6 for evaluation of overall TIL, changes in activation, proliferation, and gene expression. MultiOmyx mIF, as described above, was used to characterize the immune phenotype of tumor-infiltrating immune populations using the following markers after custom validation of the antibody panel: ICOS, PD-1, PD-L1, OX40, Foxp3, CD4, CD8, granzyme B, Ki67, CD16, CD56, HLA-DR, S100, and PanCytokeratin.

### Statistical Analysis

log-rank (Mantel–Cox test), Gehan–Breslow–Wilcoxon, one-way ANOVA, or Student *t* tests were used as specified in the figure legends. Data were analyzed with GraphPad Prism software (GraphPad, RRID:SCR_002798) or R (Supplementary Fig. S1 only). *P* values of <0.05 were considered statistically significant (*, *P*≤0.05; **, *P*≤0.01; ***, *P*≤0.005; ****, *P* < 0.0001).

### Data and Materials Availability

**FIGURE 1:**
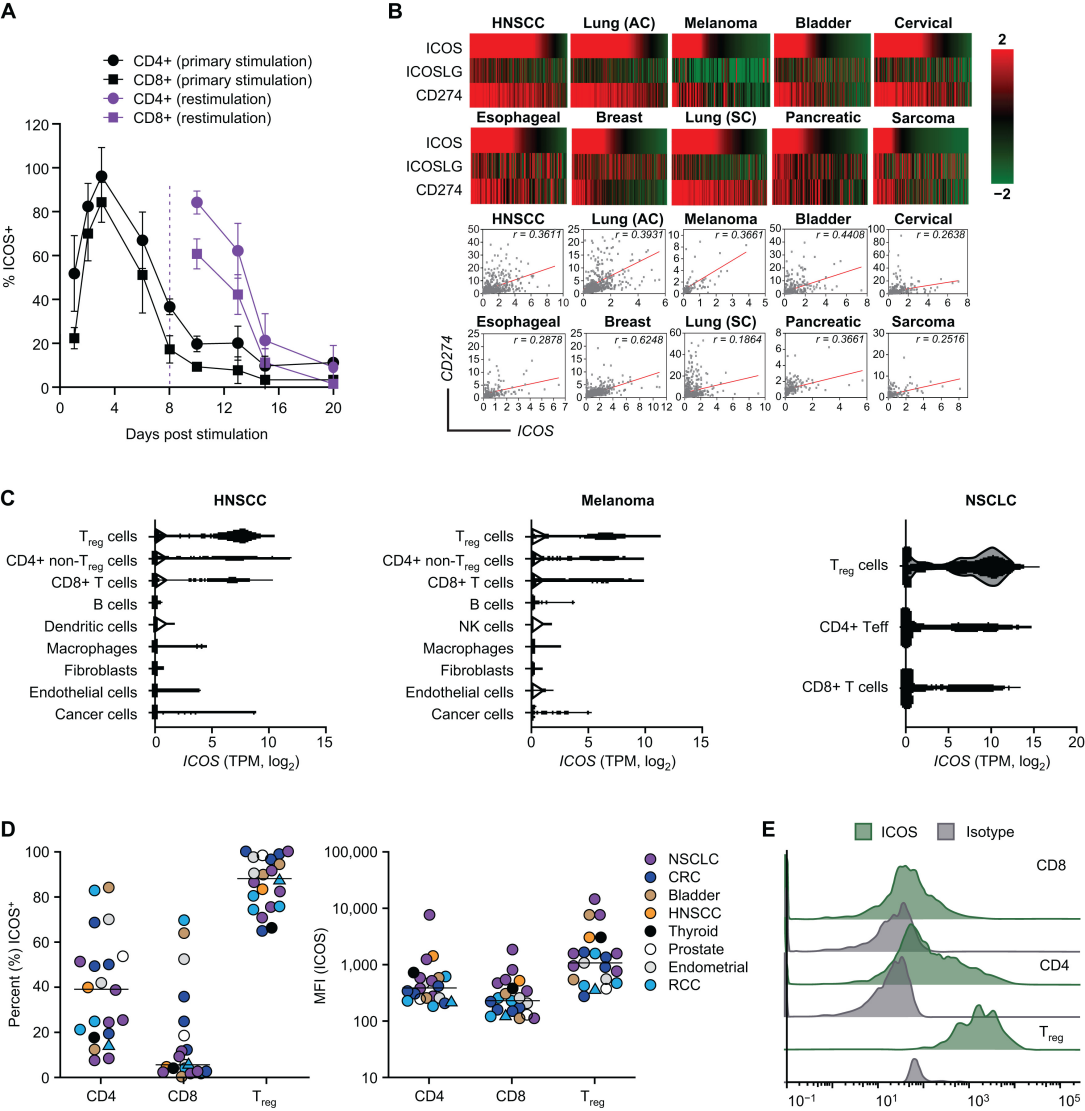
ICOS serves as a T-cell activation biomarker. **A,** Percentage expression of ICOS on T-cell subsets from healthy human donor PBMC following primary stimulation (day 0) and restimulation (day 8) using plate-coated anti-CD3/CD28 and flow cytometry (nonspecific binding was blocked with human or mouse Fc block). Data represent the mean ± SD of *n* = 4 samples; for gating strategy see Supplementary Fig. S1. **B,** TCGA-derived gene expression analysis of *ICOS*, ICOSLG (*ICOS-LG*), and PD-L1 (*CD274*) and Pearson correlation (*r*) analysis of *ICOS* versus PD-L1 (*CD274*) in different tumor types. Expression values were obtained from TCGA RNA-seq analysis. For heat map visualization, gene expression values obtained from TCGA were normalized within each indication using robust center scaling. Pearson correlation, significance, and number of tumors analyzed per indication can be found in Supplementary Table S5. Tumors were sorted on the basis of ICOS mRNA expression values from high to low. **C,** Violin plots showing expression of *ICOS* (log_2_ TPM) in various T-cell subsets from HNSCC, melanoma, and NSCLC RNA-seq data sets (26–28). **D** and **E,** ICOS expression on freshly dissociated patient TILs using flow cytometry (nonspecific binding was blocked with human or mouse Fc block). **D,** Mean fluorescence intensity (log_10_ MFI) of ICOS on individual T-cell populations. Median ICOS expression for each population is indicated by the horizontal bar with each symbol representing an individual patient (*n* = 1–6 samples/cancer type). For raw data and gating strategy, please see Supplementary Table S2 and Supplementary Fig. S2. **E,** Histograms of ICOS expression and isotype control on tumor-infiltrating T cells from a representative patient with colorectal cancer. AC, adenocarcinoma (LUAD); CRC, colorectal cancer; HNSCC, head and neck squamous cell carcinoma; ICOS, inducible T cell costimulator; NSCLC, non–small-cell lung carcinoma; PBMC, primary human peripheral blood mononuclear cells; PD-L1, programmed death ligand-1; RCC, renal cell carcinoma; SC, squamous cell (LUSC); TCGA, The Cancer Genome Atlas; TIL, tumor-infiltrating lymphocyte.

**FIGURE 2:**
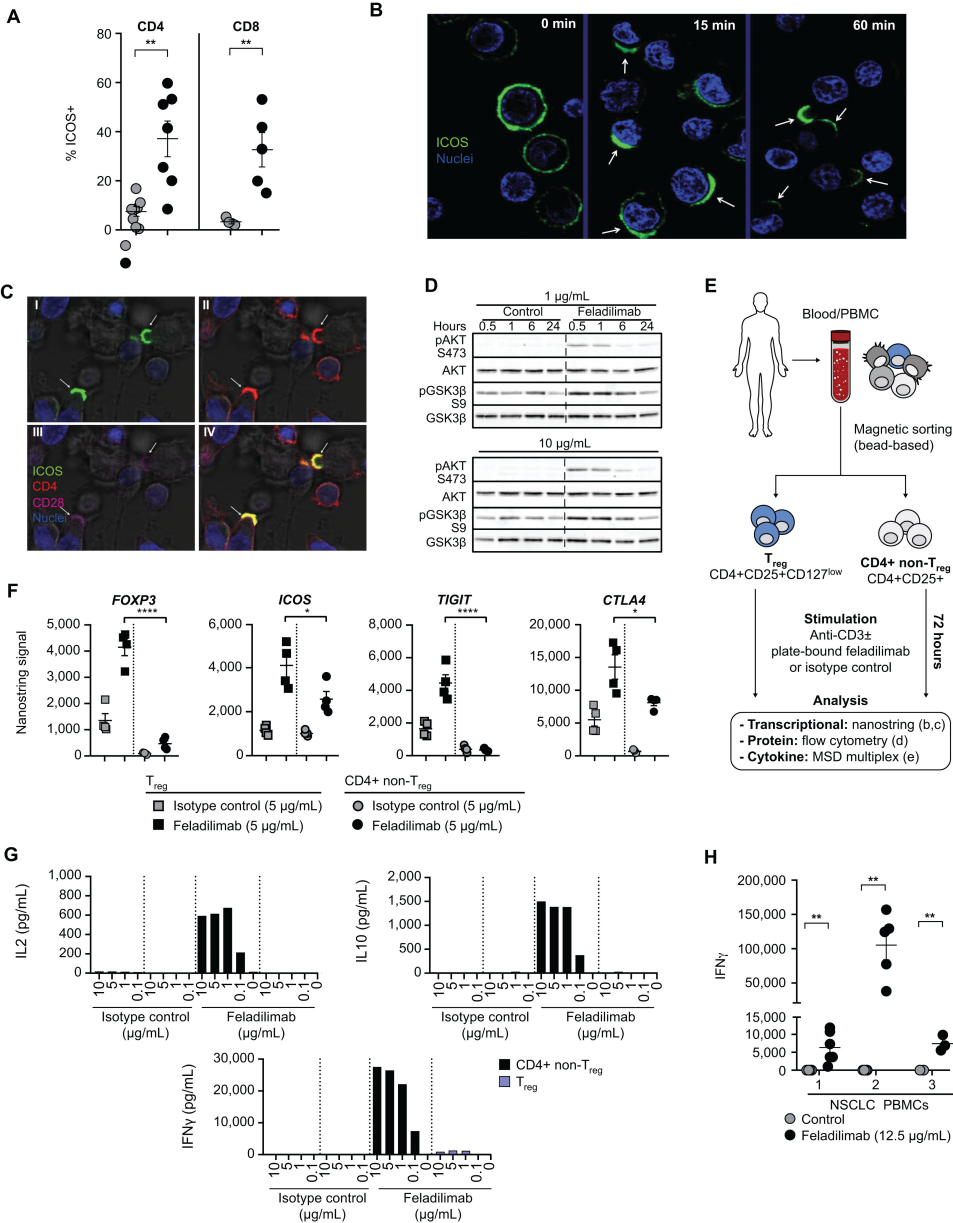
*In vitro* functional characteristics of antihuman ICOS agonist mAb feladilimab. **A,** Detection of ICOS on naïve or preactivated (48 hours anti-CD3/CD28) peripheral blood T cells from healthy human donors using feladilimab (3 μg/mL, soluble) and subsequent FITC-conjugated anti-human IgG secondary antibody. Each symbol represents an individual donor. See Supplementary Fig. S1 for flow cytometry gating strategy. Confocal microscopy illustrating kinetics of ICOS cellular localization using feladilimab (3 μg/mL, soluble) as the antibody for staining (**B**) and T cell–DC interactions following coincubation with feladilimab and CD3/CD28 (**C**). **C,** Stimulated T cells exhibit ICOS polarization and mobilization toward neighboring DCs (dark), localizing with related costimulatory receptor CD28; (I–III) denote image overlays, with (IV) combining all markers. **D,** Representative Western blot analyses of AKT pathway phosphorylation in activated CD4^+^ T cells following treatment with soluble feladilimab or isotype control (1 and 10 μg/mL, soluble) for 0–24 hours; uncropped images available in Supplementary Fig. S12A. As illustrated in **E**, CD4^+^ non-T_reg_ (CD4^+^ CD25^−^) and T_reg_ cells (CD4^+^ CD25^+^ CD127_low_) were isolated from healthy donor peripheral blood and stimulated using plate-bound anti-CD3 (1 μg/mL) ± feladilimab or isotype control (each at 5 μg/mL) for 72 hours. **F,** RNA-based analysis (Nanostring) of T_reg_-associated marker (*FOXP3*, *ICOS*, *TIGIT*, and *CTLA4*) expression by the stimulated cell subsets (each symbol represents an individual donor). **G,** Cytokine-based analysis of T-cell subsets following stimulation with plate-bound anti-CD3 and a dose range of feladilimab or isotype control; see Supplementary Fig. S1 for gating strategy. **H,** IFNγ production in the supernatant of PBMC cultures from patients with NSCLC following plate-bound feladilimab and anti-CD3 (0.6 μg/mL) stimulation (24 and 48 hours for healthy donors; 72 hours for patients with NSCLC). Data in A and H represent the mean ± s.e.m; significance determined by unpaired Student *t* test. Where shown, significance was determined by one-way ANOVA. AKT, protein kinase B; Fc, fragment crystallizable; ANOVA, analysis of variance; DC, dendritic cell; ICOS, inducible T cell costimulator; IFN, interferon; Ig, immunoglobulin; mAB, monoclonal antibody; NSCLC, non–small cell lung carcinoma; PBMC, peripheral blood mononuclear cells; s.e.m., standard error of the mean.

Raw data for Figs. 1D, 1E, 2G, 4B, 4C, and Supplementary Fig. S6C are provided as Supplementary Tables S2–S4. All requests for other data should be directed to the corresponding author and will be provided upon reasonable request when possible. All requests for raw and analyzed data and materials will be promptly reviewed by GSK to verify whether the request is subject to any intellectual property or confidentiality obligations. Patient-related data not included in the article were generated as part of clinical trials and may be subject to patient confidentiality. Any data and materials that can be shared will be released via a material transfer agreement.

## Results

### ICOS is a Robust T-cell Activation Marker and a Prognostic Biomarker in HNSCC

Primary PBMC-derived T cells were stimulated with plate-bound anti-CD3/CD28 to evaluate the kinetics of ICOS expression following T-cell activation. Consistent with the transient nature of ICOS expression, and dependence on TCR activation (32–34), the percentage of ICOS expression on CD4^+^ (includes T_reg_ cells) and CD8^+^ T cells peaked between 2 and 5 days after stimulation (or restimulation using the same antibodies; Fig. 1A; Supplementary Fig. S1).

As some level of T-cell activation may be expected in or around the tumor microenvironment (TME), we also evaluated ICOS mRNA expression in a range of cancer settings using TCGA database. We found that ICOS gene expression was enriched in several tumor indications, including HNSCC, lung carcinoma, melanoma, and bladder cancer (Fig. 1B). Consistent with immune activation, ICOS gene expression positively correlated with programmed death ligand-1 (PD-L1 or *CD274*) expression (Fig. 1B; Supplementary Table S5). As ICOS pathway activation is inferred by coexpression of ICOS and ICOS-L, ICOS, and ICOS-L (*ICOSLG*) mRNA were also analyzed in these indications (Fig. 1B). We observed a stark disconnect between ICOS and *ICOSLG* gene expression across several cancers, suggesting a potential mechanism for regulating ICOS costimulation within the TME by using an ICOS agonist mAb when ICOS-L expression is comparatively low, and underscoring the untapped potential of ICOS signaling in cancer.

To corroborate the publicly available TCGA data and evaluate cell-specific ICOS expression, we analyzed available melanoma, HNSCC, and NSCLC single-cell RNA-seq datasets (26–28). *ICOS* expression was largely restricted to TILs, with robust expression across individual T-cell subsets (Fig. 1C). Although high *ICOS* expression was observed in T_regs_, it was not a distinct feature of these, as several tumors demonstrated overlapping levels of *ICOS* expression on CD8^+^, conventional CD4^+^, and T_reg_ cell populations. Moreover, flow cytometry of freshly dissociated TILs from patients with a range of tumor types revealed similar expression patterns at the protein level on CD4^+^ and CD8^+^ T cells (Fig. 1D and E; Supplementary Fig. S2; Supplementary Table S2).

### ICOS Agonist mAb Feladilimab Binds to and Activates Primary Human T Cells

To evaluate the potential functional effects of ICOS costimulation in the setting of human disease, we generated feladilimab, a humanized mAb with an optimized Fc region with reduced potential to bind activating FcγRs. Unlike ICOS-L, which has been shown to bind to the two nearest structurally related proteins CTLA-4 and CD28 (2, 35), feladilimab selectively binds to human ICOS with high affinity, but does not bind to murine ICOS or human CD28/CTLA-4 [Supplementary Fig. S3A–S3E (murine binding data not shown)].

Consistent with our results demonstrating ICOS expression on activated T cells (Fig. 1A), feladilimab exhibited binding to CD4^+^ and CD8^+^ T cells from anti–CD3/CD28-activated PBMC from human donors (Fig. 2A; Supplementary Fig. S1). To further examine the expression kinetics, dynamics, and cellular localization of ICOS by primary activated human CD3^+^ T cells following exposure to feladilimab, time-lapse imaging was performed using confocal microscopy. Within minutes, stimulated T cells exhibited cell-surface ICOS polarization, the mobilized T cells scanned the culture until binding with a DC was initiated and ICOS accumulated at the point of contact (Fig. 2B; Supplementary Movie S1). Furthermore, feladilimab-induced ICOS complexes rapidly colocalized with CD28 to facilitate immune synapses between T cells and APCs (Fig. 2C). These results indicate that ICOS costimulation following T-cell activation induces T-cell mobilization and receptor polarization at the immune synapse.

ICOS has previously been shown to activate AKT in human T cells following engagement of ICOS-L (36). Accordingly, addition of feladilimab to anti–CD3/CD28-activated primary human CD4^+^ T cells elicited an increase in AKT and GSK3β phosphorylation (Fig. 2D; Supplementary Fig. S4A). This observation was corroborated using Ba/F3 cells engineered to express human ICOS (Supplementary Figs. S4B, S4C, S5A, and S5B), highlighting the potential role of the PI3K pathway in antibody-mediated ICOS costimulation.

When used in a plate-bound format to mimic *in vivo* Fc-FcγR cross-linking, feladilimab elicited downstream functional effects in primary human TCR-activated PBMC, including induction of CD69 and increased proliferation (determined via Ki67 staining) of both CD4^+^ and CD8^+^ T cells (Supplementary Figs. S1, S6A, and S6B). Notably, we found that while plate-bound feladilimab elicited stronger costimulatory responses, soluble feladilimab was also able to potentiate immune activation (Fig. 2D; Supplementary Fig. S4A and S7). Treatment of isolated human CD3^+^ T cells with feladilimab also led to a significant increase in the expression of Th1-associated transcription factors T-bet (*TBX21*) and granzyme-B (*GZMB*), as well as a marked decrease in L-selectin (*SELL*) expression (Supplementary Fig. S6C; Supplementary Table S3), signifying a transition toward an effector phenotype (37) and increased potential for antitumor activity. Furthermore, feladilimab enhanced the production of IFNγ, IL17, IL10, and TNFα by TCR-activated healthy donor and cancer patient PBMC (Fig. 2H; Supplementary Figs. S5C, S5D, and S6D). Increases in production were highest for IL17, followed by IFNγ and TNFα, while production of other cytokines was only modestly (or inconsistently) induced (Supplementary Fig. S5C and S5D). Interestingly, feladilimab exhibited differential activity on isolated human CD4^+^ non-T_reg_ cells versus T_reg_ cells (Fig. 2; Supplementary Fig. S6). Transcriptional and cytokine-based analysis revealed that while many of the commonly associated T_reg_ cell markers (e.g., *FOXP3*) and various activation markers (e.g., *CD25*) were maintained following *in vitro* stimulation, feladilimab induced dramatic changes in CD4^+^ non-T_reg_ cell functional markers [e.g., granzyme A and B (GZMA and GZMB)] and cytokine production (e.g., IFNγ and IL17; Fig. 2F and G; Supplementary Figs. S1 and S6E–S6G).

### ICOS Agonist mAbs Demonstrate Single-agent Antitumor Activity in Mouse Tumor Models

**FIGURE 3:**
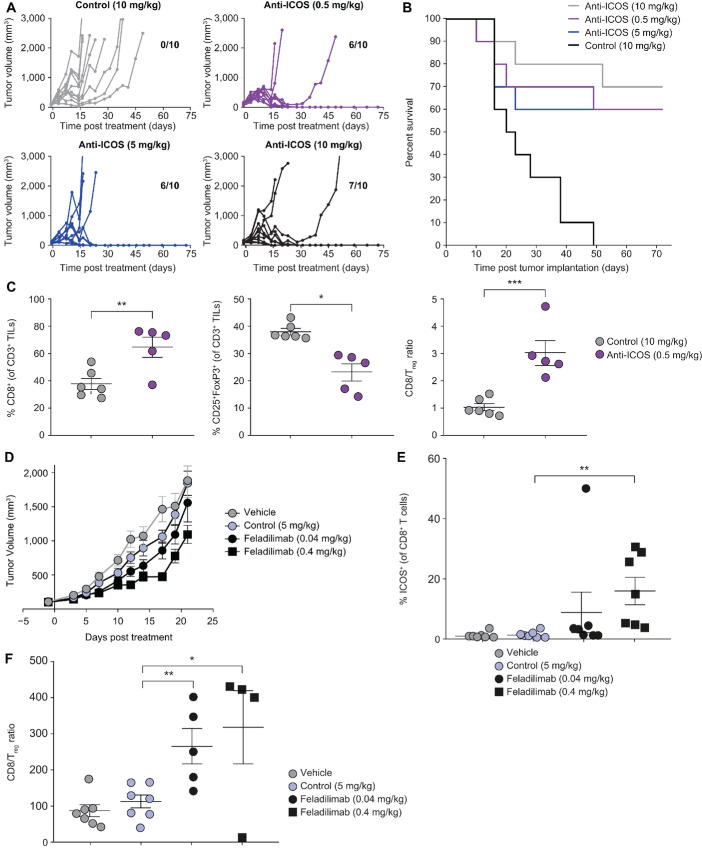
ICOS agonist mAbs demonstrate single-agent antitumor activity. **A–C,** EMT6 (subcutaneous) tumor-bearing BALB/c mice were administered intraperitoneally biweekly with anti-mouse ICOS mAb [7E.17G9 (mIgG1)] or isotype control (mIgG1) for a total of six doses and evaluated for tumor growth (**A**), survival (**B**), and pharmacodynamic changes (**C**) within tumors. Each line in A represents an individual mouse (*n* = 10/group). Number of tumor-free mice at study termination are indicated within each subpanel. **B,** Kaplan–Meier plot illustrating OS in A. **C,** Percentage of tumor-infiltrating CD8^+^ T cells, T_reg_ cells, and associated CD8:T_reg_ cell ratio 48 hours after the third dose of anti-ICOS mAb (10 μg, ∼study day 7) assessed using flow cytometry (nonspecific binding was blocked with human or mouse Fc block). Each symbol represents an individual mouse. **D–F,** A549 (subcutaneous) tumor-bearing NSG mice were administered intraperitoneally biweekly with feladilimab or isotype control for a total of six doses. **D,** A549 tumor growth kinetics for controls (PBS or isotype) and feladilimab-treated groups (0.04 mg/kg, black; 0.4 mg/kg, red). Percentage of tumor-infiltrating ICOS+ CD8^+^ T cells (**E**; assessed using flow cytometry with nonspecific binding blocked using human or mouse Fc block) and associated CD8:T_reg_ cell ratio (**F**) 48 hours after the fourth dose of feladilimab (∼study day 21). Data in C, D, E, and F represent the mean ± s.e.m. Significance was determined by unpaired Student *t* test. See Supplementary Fig. S1 for flow cytometry gating strategy for C, E, and F. CD8/Treg ratios were calculated on the basis of percent (%) positive CD8s (of live CD45^+^) and FoxP3/CD25^+^ Treg cells (of live CD45^+^ CD4^+^ cells). ICOS, inducible T-cell costimulator; i.p., intraperitoneal injection; mAb, monoclonal antibody; OS, overall survival; s.e.m., standard error of the mean.

To evaluate the antitumor potential of ICOS agonism, we treated EMT6 (syngeneic breast cancer model) tumor-bearing mice with an anti-mouse ICOS mAb [an Fc variant of 7E.17G9 mouse (m)IgG1]. To better parallel the Fc-Fcγ receptor biology of feladilimab, 7E.17G9 was grafted on an Fc region with reduced potential for coengagement with activating FcγR (mIgG1). Effective tumor control was observed in mice administered with 7E.17G9 at a range of doses associated with clinically tolerable exposure (0.5, 5, and 10 mg/kg; Fig. 3A and B). Plasma concentrations of the mIgG1 anti-ICOS mAb were dose dependent and were quantifiable during the entire study period at doses ≥0.5 mg/kg (Supplementary Fig. S8). Consistent with the mechanism of ICOS-mediated costimulation and effector expansion, anti-ICOS treatment elicited a significant increase in CD8^+^ TILs together with a decrease in the percentage of T_reg_ cells in the TME, resulting in a dramatic shift in the CD8:T_reg_ cell ratio (Fig. 3C; Supplementary Fig. S1). To assess the therapeutic activity of feladilimab *in vivo,* NSG mice bearing human NSCLC A549 tumors were engrafted intravenously with human PBMCs once tumors were approximately 100 mm^3^. At a dose of 0.4 mg/kg, feladilimab demonstrated significant tumor control when compared with control (Fig. 3D). Although the relative contributions of alloreactive or tumor-specific T cells to tumor control in PBMC-engrafted models are difficult to determine, the results suggest an overall enhancement of the T-cell response following exposure to feladilimab and are further supported by antitumor responses observed in a CD34^+^ HPSC-engrafted patient-derived xenograft model of breast cancer (BR1126; Supplementary Fig. S9). With respect to target engagement, feladilimab exhibited dose-dependent receptor occupancy (Fig. 3E; Supplementary Fig. S1) in A549 tumor-bearing mice and, comparable with our observations with the mIgG1 anti-ICOS mAb in EMT6 tumor-bearing mice (Fig. 3C), significantly increased the CD8:T_reg_ cell ratio in the tumor (Fig. 3F; Supplementary Fig. S1).

### ICOS Stimulation and PD-1 Inhibition Demonstrate Pathway Interplay and Combine to Improve Antitumor Activity in Mice

Given the role of ICOS signaling in the expansion and improved function of recently activated T cells (38), feladilimab is expected to have increased antitumor activity in combination with immunotherapies that prime or modulate the immune system, such as anti-PD-1 agents. We therefore evaluated the reciprocity between modulation of PD-1 and ICOS and their respective expression patterns.

**FIGURE 4:**
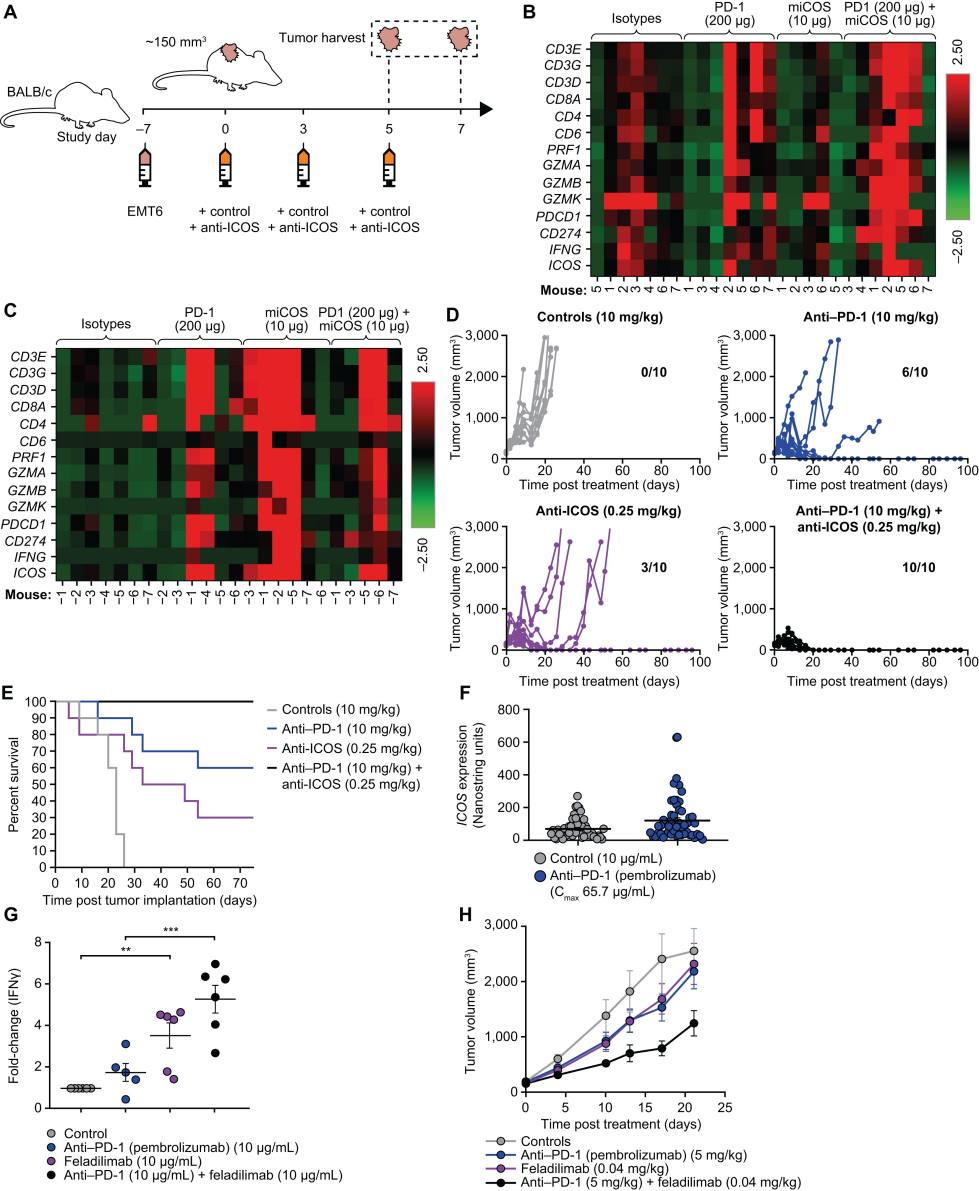
ICOS agonist mAbs demonstrate improved antitumor activity in combination with PD-1 blockade. EMT6 (subcutaneous) tumor-bearing BALB/c mice were administered intraperitoneally biweekly with anti-mouse ICOS mAb [7E.17G9 (mIgG1)], anti-PD-1 mAb (RMP1-14), or isotype controls (mIgG1 and rat IgG2a, respectively) alone and in combination for a total of six doses. Mice were evaluated for pharmacodynamic changes (**B** and **C**) within tumors, tumor growth (**D**), and survival (**E**). As illustrated in **A**, transcriptional analysis was performed (*n* = 5–7) on tumor tissue harvested from mice 48 hours after second (**B**) and third doses (**C**) of indicated mAbs; raw data in Supplementary Table S4. Each line in **D** represents an individual mouse (*n* = 10/group). Tumor-free mice at study termination are indicated within each subpanel. **E,** Kaplan–Meier plot illustrating OS in D. **F,***ICOS* expression following *ex vivo* anti–PD-1 (pembrolizumab) or vehicle control treatment of tumor slices from patients with HNSCC for 48 hours. Each symbol represents an individual human tumor sample (*n* = 50/group). **G,** Fold change in IFNγ production by TILs from dissociated NSCLC tumor samples (*n* = 5–6 samples/group) following exposure to anti-CD3 (plate-bound, 0.6 μg/mL) in concert with anti-PD-1 (pembrolizumab, soluble) or feladilimab (plate-bound) alone or in combination for 24 hours. **H,** A2058 (subcutaneous) tumor-bearing NSG mice were administered intraperitoneally biweekly with feladilimab and anti-PD-1 (pembrolizumab) alone or in combination for a total of six doses and assessed for tumor growth inhibition (*n* = 10/group). Data in F–H are represented as mean ± s.e.m. Significance in G determined by unpaired Student *t* test. Despite trends in tumor growth kinetics following combination treatment, the curves in H were not significantly different as determined by one-way ANOVA. ANOVA, analysis of variance; HNSCC, head and neck squamous cell carcinoma; ICOS, inducible T cell costimulator; IFN, interferon; i.p., intraperitoneal injection; mAb, monoclonal antibody; NSCLC, non–small cell lung carcinoma; OS, overall survival; PD-1, programmed cell death protein 1; s.e.m., standard error of the mean; TIL, tumor-infiltrating lymphocyte.

To test whether a PD-1 blocking antibody could augment the antitumor activity of an ICOS agonist mAb, we administered EMT6 tumor-bearing mice with the mIgG1 ICOS agonist mAb (7E.17G9) alone and in combination with anti-PD-1 antibody (clone RMP1-14) via intraperitoneal injection. Transcriptional analysis was conducted on tumor tissue harvested 48 hours after second and third doses of control or therapeutic mAbs (Fig 4A–C). Notably, while broad transcriptional changes were noted early (day 5; Fig. 4B; Supplementary Table S4), anti-ICOS mAb (mIgG1 or rIgG2b) treatment significantly induced expression of both PD-1 (*PDCD1*) and PD-L1 (*CD274*) in tumor tissue on day 7 (Fig. 4C; Supplementary Fig. S10A; Supplementary Table S4). Similarly, increases in *ICOS* expression were observed following treatment with the anti-PD-1 mAb (Fig. 4B and C; Supplementary Fig. S10B; Supplementary Table S4). Alongside changes in target expression, significant increases in serum IFNγ levels and T-cell activation/function in tumor-draining lymph nodes were observed following treatment with the mIgG1 anti-ICOS mAb and the anti-PD-1 mAb combined (Supplementary Fig. S10C and S10D). Combination therapy resulted in a dramatic improvement in tumor rejection and a significant increase in long-term survival relative to monotherapies (Fig. 4D and E).

Analysis by multiplex IHC showed that ICOS and PD-1 are often coexpressed on CD3^+^ cells in the TME in different tumor types. PD-1 and ICOS coexpression was particularly noteworthy in HNSCC, but was also observed in esophageal cancer, lung cancer, and melanoma (Supplementary Fig. S10E). Comparatively, low coexpression was seen in normal tissues (Supplementary Fig. S10E), supporting the rationale for combining ICOS agonism with PD-1 blockade in the highlighted indications. *Ex vivo* treatment of tumor tissue from patients with HNSCC with pembrolizumab significantly increased upregulation of *ICOS* gene expression relative to untreated controls (Fig. 4F). Reciprocally, treatment of PBMC from patients with NSCLC, HNSCC, and melanoma with feladilimab upregulated PD-1 expression on CD4^+^ and CD8^+^ T cells (Supplementary Figs. S1 and S10F).

These findings were extended to a primary dissociated tumor assay, wherein tumor samples from patients with NSCLC were enzymatically dissociated and expanded TILs were stimulated with plate-bound anti-CD3 and exposed to feladilimab, pembrolizumab, or the combination. While ICOS costimulation alone resulted in a significant increase in IFNγ compared with control, the combination of feladilimab and pembrolizumab induced a significantly greater fold change in IFNγ production than pembrolizumab alone (Fig. 4G), consistent with results with activated human PBMC (Supplementary Fig. S10G). Although there was a trend toward increased IFNγ with the combination of feladilimab and pembrolizumab versus ICOS costimulation alone, the difference was not significant.

Finally, we extended these observations to a humanized murine model, wherein human PBMC-engrafted NSG mice bearing A2058 human malignant melanoma tumors were administered feladilimab and pembrolizumab. While both feladilimab and PD-1 blockade demonstrated modest monotherapeutic effects, the combination of feladilimab and pembrolizumab resulted in enhanced tumor control relative to the monotherapies, though this was not statistically significant (Fig. 4H). Collectively, these results highlight the complementarity of ICOS costimulation and PD-1 blockade in both rodent and human disease settings.

### Clinical Evaluation of ICOS Stimulation Using Feladilimab

On the basis of the nonclinical evidence supporting the utility of anti–ICOS-based costimulation to promote antitumor responses, we initiated an ongoing first-in-human clinical trial (INDUCE-1; NCT02723955) to translate these findings to patients with advanced solid tumors in the clinic. The study was designed to evaluate the safety/tolerability, pharmacokinetics, pharmacodynamics, and preliminary clinical activity of feladilimab alone and in combination with other anticancer agents, including pembrolizumab. A range of feladilimab doses as a monotherapy and in combination with pembrolizumab (200 mg once every 3 weeks) were being investigated in the dose-escalation phase of the trial, and the dose-expansion phase is further evaluating feladilimab at doses selected in the dose-escalation phase.

**FIGURE 5:**
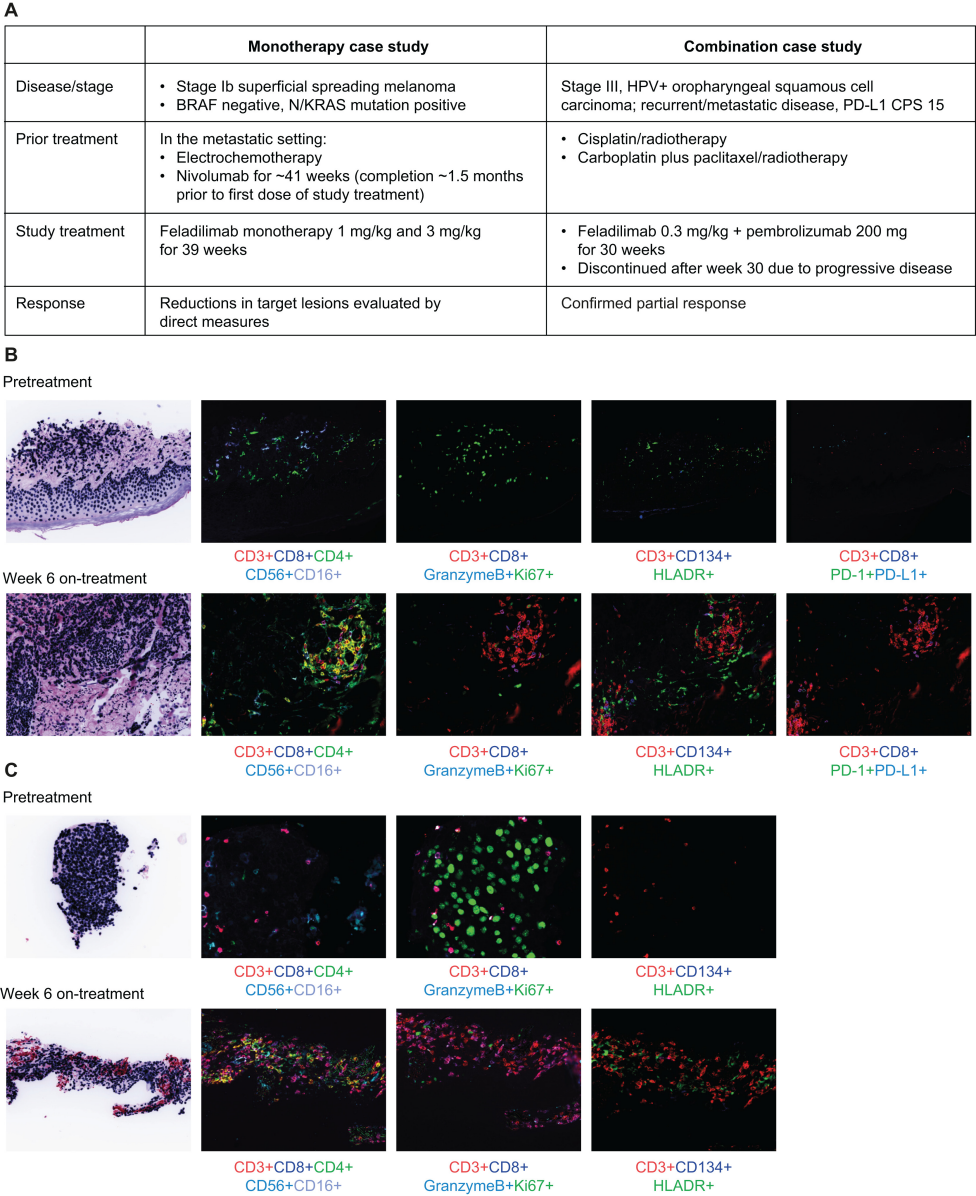
Clinical patient case studies of feladilimab monotherapy and in combination with pembrolizumab. **A,** Clinical history and treatment details for a patient with melanoma who received feladilimab monotherapy and a patient with oropharyngeal squamous cell carcinoma who received feladilimab in combination with pembrolizumab. Tumor sample immunohistochemistry and expression of markers for TIL activation, cytotoxic function and proliferation for the monotherapy case study (**B**) and the combination therapy case study (**C**). See Supplementary Fig. S11B for CT imaging of tumor lesions for the patient with oropharyngeal squamous cell carcinoma. CPS, combined positive score; CT, computed tomography; HLA-DR, human leukocyte antigen-DR; HPV, human papillomavirus; PD-L1, programmed death ligand-1; TIL, tumor-infiltrating lymphocyte.

Pharmacodynamic and tumor response analyses using immune-related and RECIST v1.1 assessments from patient case studies from INDUCE-1 provided proof-of-concept support for the clinical evaluation of an ICOS agonist as monotherapy and in combination with PD-1 blockade (Fig. 5; Supplementary Table S6; Supplementary Figs. S11 and S12); for example, in an anti-PD-1–experienced patient with metastatic melanoma, mIF on tumor biopsies taken before and after feladilimab-treatment (1 and 3 mg/kg; Fig. 5A). We found that ICOS costimulation with feladilimab elicited an increase in TILs from baseline (pretreatment), including cytotoxic T cells, Th cells, and (natural killer) natural killer cells, and a reduction in proliferating tumor cells (Fig. 5B). As evidenced by increased granzyme B+ T cells and elevated PD-1, PD-L1, and HLA-DR, enhanced activation of TILs was also observed (Fig. 5B). Notably, these effects were associated with reductions in target lesions in this patient (Fig. 5A). Similar activity following treatment with feladilimab monotherapy has been observed in other patients with anti-PD-1/L1 treatment-experienced cancers in INDUCE-1, including melanoma and HNSCC indications; these additional example case studies include a patient with relapsed HNSCC who attained a partial response (PR; ref. 39) and a patient with metastatic nodular melanoma who had high burden of disease and attained a PR followed by stable disease over the 48-week treatment course (Supplementary Fig. S11A and S11B; ref. 40), with corresponding reductions in lung and arm metastases observed from baseline to week 45 (Supplementary Fig. S12A). In addition, in an example case study is a patient with stage III HPV+ oropharyngeal squamous cell carcinoma treated with feladilimab at 0.3 mg/kg in combination with a flat dose of pembrolizumab 200 mg once every 3 weeks (Fig. 5A), who showed progressive reduction in lung metastases from baseline to week 27 (Supplementary Fig. S12B) and changes in tumor-infiltrating T-cell functional markers (Fig. 5C), consistent with those observed with feladilimab monotherapy (Fig. 5B).

## Discussion

Immune checkpoint inhibitors have transformed the practice of clinical oncology; yet, there remains a need for improvement in clinical outcomes by rational development of novel therapeutic approaches to overcome the multifaceted mechanisms of tumor evasion. The nonclinical evidence we present provides support for ICOS stimulation as a promising therapeutic strategy in the context of cancer.

Because of priming of T cells through contact with cognate antigens and activation is necessary for induction of ICOS expression (7), ICOS-mediated T-cell costimulation is expected to be most active in disease settings where an antitumor immune response is primed, either as an inherent feature or by prior lines of therapy. Antibody-mediated ICOS costimulation is anticipated to have monotherapeutic activity in these contexts. However, the prognostic impact of ICOS expression and pathway activation in different tumor types can vary, highlighting the complexity of ICOS biology, and immune dynamics in general, in cancer (13, 41). We observed antitumor activity in nonclinical models, accompanied by increases in the CD8:T_reg_ ratios. While the increase in ratios could reflect differences in tumor size or other factors, it suggests that ICOS agonism can provide therapeutic benefit despite the presence of ICOS-positive T_reg_ cells. Consistent with this observation, we found that *in vitro* antibody-mediated ICOS stimulation resulted in preferential induction of cytokines in CD4^+^ non-T_reg_ cells relative to T_reg_ cells. Nevertheless, strategies for the clinical development of ICOS agonist mAbs should closely evaluate the basal differences in T-cell subtypes and functional status across indications of interest, in particular tumor types known to exhibit innate or therapy-induced priming of antitumor immunity, such as HNSCC and metastatic melanoma, respectively.

Feladilimab was primarily identified on the basis of the mechanism to potentiate primary T-cell activation. In addition to demonstrating primary T-cell activation via induction of cytokines and phenotypic markers of activation, we demonstrated antibody-mediated forward signaling via AKT phosphorylation. Typically, experiments with primary T cells require cross-linking by either secondary antibody or cell-mediated FcrγIIB-Fc coengagement. However, as shown here, detectable antibody-mediated activation may be observed without strong cross-linking (e.g., in solution vs. plate-bound antibody) as reported previously (38). Nuances in anti-ICOS forward signaling and the associated impact of Fc biology will be characterized in more detail in a future publication.

Intuitively, more effective antitumor immunity could potentially be achieved through combination of ICOS activation with other agents known to elicit T-cell activation via distinct mechanisms or pathways. The observed, robust induction of IFNγ by feladilimab in *ex vivo* and *in vivo* studies, in addition to upregulation of PD-1 and PD-L1, supports the rationale behind combining ICOS agonism with PD-1 blockade, with IFNγ known to act on negative feedback by upregulation of PD-L1 (42). Conversely, anti-PD-1 treatment induced expression of ICOS on CD4^+^ and CD8^+^ T cells. The combination of feladilimab and the PD-1 blocking antibody pembrolizumab increased both proinflammatory cytokine production *ex vivo* (dissociated NSCLC tumors) and antitumor activity in a humanized mouse tumor model (A2058), providing additional rationale to evaluate the combination clinically. These observations have thus far shown some level of translatability to antitumor effects in PD-1/L1-experienced patients (e.g., metastatic melanoma) and a promising signal in combination with pembrolizumab in select patients with PD-1/L1–naïve HNSCC, with an overall response rate of 24%, and a disease control rate ≥18 weeks of 47% (43). The combination regimen has shown durable responses in patients with PD-1/L1–naïve HNSCC, with all responding patients maintaining benefit for ≥6 months (43). Furthermore, previously reported preliminary safety data have demonstrated that feladilimab alone and in combination with pembrolizumab has a manageable safety profile in patients with advanced solid tumors at a dose range of 0.001–3 mg/kg feladilimab, with grade ≥3 treatment-related adverse events observed in approximately 5%–11% of patients (39, 40, 43). However, more work is needed to identify relevant patient populations, particularly due to the depth of heterogeneity in cancer patient characteristics (e.g., tumor-immune contextures, genetic predisposition, etc.).

Other monoclonal ICOS antibodies such as alomfilimab (KY1044) and vopratelimab (JTX-2011) have demonstrated agonistic activity on human primary T cells, with requirement for FcR cross-linking, induction of proinflammatory cytokines such as IFNγ, and activation of forward signaling pathways; they have also shown antitumor efficacy as monotherapies in mouse models, with improved efficacy when administered in combination with anti-PD-1 antibodies (44, 45). No direct comparisons of feladilimab with these antibodies have been performed; however, the responses seen with the differences in Fc and epitope variations are considered favorable for feladilimab.

Our data represent the foundation for evaluating a humanized IgG4 ICOS agonist mAb, feladilimab, in clinical trials. In case studies from INDUCE-1 (NCT02723955), evidence of ICOS receptor engagement and immune activation with feladilimab treatment was observed in different tumor types as evidenced by the changes in the TME of patients with melanoma and HNSCC who experienced some clinical benefit and the overall reductions in tumor burden. While these example cases represent the translatability of functional changes with ICOS stimulation in patients who had clinical benefit, these findings may not characterize the cumulative data from all patients treated with feladilimab. Although the presented data from INDUCE-1 study are preliminary, these data support the randomized investigation of feladilimab in combination with pembrolizumab; however, it should be acknowledged that the INDUCE-3 and INDUCE-4 studies investigating the same combination in participants with recurrent or metastatic HNSCC were halted. Analysis is underway, and this will be the subject of a future article (46). Importantly, as the clinical data are limited in terms of understanding the pharmacodynamic effects of ICOS stimulation and the interactions of ICOS stimulation with immune checkpoint inhibitors, it will be critical to determine the tumor-immune conditions necessary to facilitate responses to ICOS stimulation that result in antitumor effects. Furthermore, similar to the clinical observations made with CTLA-4 and PD-1 targeting agents, clinical responses with ICOS per RECIST or progression-free survival may be unreliable endpoints that may indicate clinical activity but require survival data for understanding of its clinical effects. Both CTLA-4 and PD-1 blocking antibodies exert their clinical benefit mostly by extending patient survival (3).

Collectively, while these data provide support for ICOS stimulation and PD-1 blockade as a therapeutic strategy for cancer, they also underscore the need to continuously refine our understanding of the dynamic TME and the translational efforts that accompany drug development to identify disease indications and patients who could benefit from these novel therapies, and the need for randomized survival data to characterize their clinical benefit potential.

## Supplementary Material

Supplementary Movie 1 Supplementary Movie 1

Supplementary Movie 1 Legend Expression dynamics and cellular localization of ICOS.

Supplementary Methods Supplementary Methods

Supplementary Figure 1 Exemplar flow cytometry gating strategy.

Supplementary Figure 2 Flow cytometry gating strategy for ICOS on individual T cell populations.

Supplementary Figure 3 Affinity and kinetics

Supplementary Figure 4 Uncropped gel images

Supplementary Figure 5 Feladilimab induces phospho-AKT in ICOS-expressing Ba/F3 cells and cytokine production in PBMC from healthy donors and cancer patients.

Supplementary Figure 6 Feladilimab-mediated ICOS co-stimulation results in differential phenotypic changes and cytokine production in CD4+ non-Treg and Treg populations.

Supplementary Figure 7 Healthy human donor CD4+ T cells were pre-activated with antiCD3/CD28 for 48 hours and then re-stimulated with plate-bound anti-CD3 in the presence of increasing concentrations of soluble or plate-bound feladilimab or isotype control for 72 hours. IFNg levels were then assessed from cell-free supernatants using Meso Scale Discovery (MSD)-based detection.

Supplementary Figure 8 Pharmacokinetic analysis of the mIgG1 anti-ICOS antibody (7E.17G9) in the peripheral blood of EMT6 tumor-bearing mice.

Supplementary Figure 9 Antitumor activity of feladilimab in a humanized patient-derived xenograft model of triple-negative breast cancer.

Supplementary Figure 10 Rationale for combination of feladilimab with anti–PD-1 blockade.

Supplementary Figure 11 Clinical case study of feladilimab monotherapy.

Supplementary Figure 12 Clinical case studies of feladilimab monotherapy and in combination with pembrolizumab.

Supplementary Table 1 Antibodies used for in vitro and in vivo assays, and experimental model

Supplementary Table 2 Table S2

Supplementary Table 3 Gene expression in human T-cell

Supplementary Table 4 Table S4

Supplementary Table 5 Pearson correlation (r) analysis of ICOS versus PD-L1 (CD274) in different tumor type.

Supplementary Table 6 Example TIL marker expression phenotypes from clinical patient case studies of feladilimab monotherapy and in combination with pembrolizumab.
