# Supplementary material for: Activating Inducible T-cell Costimulator Yields Antitumor Activity Alone and in Combination with Anti-PD-1 Checkpoint Blockade
Source: Cancer Res Commun. 2023 Aug 16;3(8):1564–79. doi: 10.1158/2767-9764.CRC-22-0293 (PMC10430783; doi:10.1158/2767-9764.CRC-22-0293)
Supplement: Supplementary Movie 1 Legend — Expression dynamics and cellular localization of ICOS. [file crc-22-0293-s02.pdf]

**Supplementary Movie 1. Expression dynamics and cellular localization of ICOS.** Human T cells were pre-stimulated with anti-CD3 for 48 hours and added to a co-culture with human dendritic cells. ICOS (green fluorescence) expression kinetics and cellular localization were monitored using AlexaFluor 488-labeled feladilimab. The movie represents time points 0–30 minutes.
