# Supplementary material for: Activating Inducible T-cell Costimulator Yields Antitumor Activity Alone and in Combination with Anti-PD-1 Checkpoint Blockade
Source: Cancer Res Commun. 2023 Aug 16;3(8):1564–79. doi: 10.1158/2767-9764.CRC-22-0293 (PMC10430783; doi:10.1158/2767-9764.CRC-22-0293)
Supplement: Supplementary Methods [file crc-22-0293-s03.pdf]

Supplementary Methods

Surface plasmon resonance and bio-layer interferometry

To evaluate feladilimab or recombinant human inducible T cell co-stimulator-ligand (ICOS-L) (his-tagged, Sino Biologica) affinity for human ICOS, surface plasmon resonance (SPR) analysis was performed using Biacore (GE Healthcare). Recombinant human ICOS was generated in-house as a rabbit Fc fusion protein and purified via protein A from human embryonic kidney cell (HEK) (The European Collection of Authenticated Cell Cultures) supernatants. Feladilimab (2.5 μg/ml) was captured on a CM5 chip using antihuman immunoglobulin G (IgG). To prevent nonspecific binding of rabbit Fc (recombinant ICOS), residual antihuman IgG on the chip surface was blocked using 0.1 mg/ml human IgG1 (hIgG1) control. Subsequently, recombinant human ICOS was passed over the captured antibodies at 256, 64, 16, 4, and 1 nM at 25ºC. For ICOS-L binding, recombinant human ICOS was first captured on the surface of a protein A chip. ICOS-L was passed over the ICOS-captured chip at 25ºC using concentrations of 256, 64, 16, 1, and 0.25 nM. Between runs, the chip surface was regenerated using a buffer containing 3M $MgCl_2$. Data were fitted to a 1:1 model using T200 evaluation software (GE Healthcare).

For bio-layer interferometry (BLI) of feladilimab binding to recombinant human CTLA-4 and CD28 (both Fc-tagged, R&D Systems), the Octet RED384 BLI system (ForteBio) was used. Feladilimab (hIgG1 Fc-variant, at 50 μg/ml) was first captured using Anti-Human IgG Fc Capture (AHC) biosensors (ForteBio) for 3 minutes at 25ºC. Prior to kinetic characterization, the individual biosensors were blocked offline for 15–30 minutes with rabbit and human IgG to prevent nonspecific binding of recombinant ICOS, cytotoxic T-lymphocyte associated protein-4 (CTLA-4), and CD28. Subsequently, the loaded/blocked sensors were used for binding analysis in the following order: rabbit IgG block (300 nM for 3 minutes), human CTLA-4 or CD28 (300 nM for 3 minutes), phosphate-buffered saline (PBS)–based buffer (for 1 minute), human or cynomolgus monkey (in-house, generated as hICOS) ICOS (256, 64, 16, 4, and 1 nM for 3 minutes), and dissociation buffer. A 1:1 kinetics model inherent to Octet analysis 7.0 software (ForteBio) was used to fit data.
