# Supplementary material for: Activating Inducible T-cell Costimulator Yields Antitumor Activity Alone and in Combination with Anti-PD-1 Checkpoint Blockade
Source: Cancer Res Commun. 2023 Aug 16;3(8):1564–79. doi: 10.1158/2767-9764.CRC-22-0293 (PMC10430783; doi:10.1158/2767-9764.CRC-22-0293)
Supplement: Supplementary Figure 1 — Exemplar flow cytometry gating strategy. [file crc-22-0293-s04.pdf]

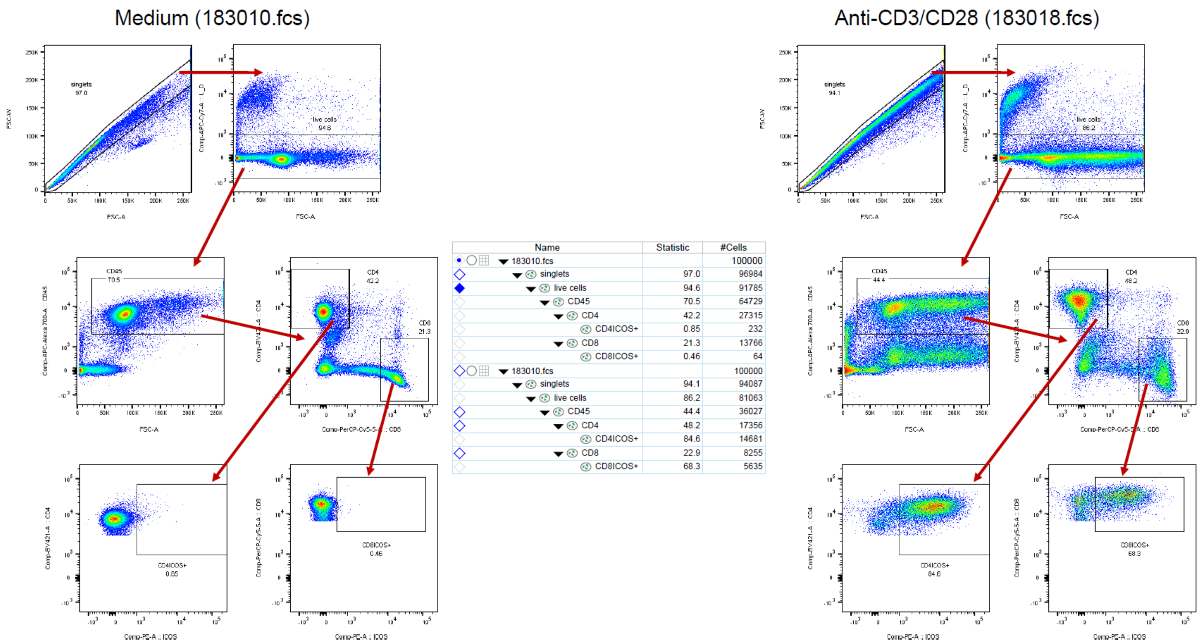

Name	Statistic	#Cells
183010.fcs		100000
singlets	97.0	96984
live cells	94.6	91785
CD45	70.5	64729
CD4	42.2	27315
CD4ICOS+	0.85	232
CD8	21.3	13766
CD8ICOS+	0.46	64
183010.fcs		100000
singlets	94.1	94087
live cells	86.2	81063
CD45	44.4	36027
CD4	48.2	17356
CD4ICOS+	84.6	14681
CD8	22.9	8255
CD8ICOS+	68.3	5635

Supplementary Fig. 1. Exemplar flow cytometry gating strategy. Associated with **Fig. 1a**, **Fig. 2a**, e, f, **Fig. 3c**, e, f, **Supplementary Fig. 6f** and **Supplementary Fig. 10f**. Example shown for ICOS.
