# Supplementary material for: Activating Inducible T-cell Costimulator Yields Antitumor Activity Alone and in Combination with Anti-PD-1 Checkpoint Blockade
Source: Cancer Res Commun. 2023 Aug 16;3(8):1564–79. doi: 10.1158/2767-9764.CRC-22-0293 (PMC10430783; doi:10.1158/2767-9764.CRC-22-0293)
Supplement: Supplementary Figure 2 — Flow cytometry gating strategy for ICOS on individual T cell populations. [file crc-22-0293-s05.pdf]

ICOS+ CD4 Gating Strategy:

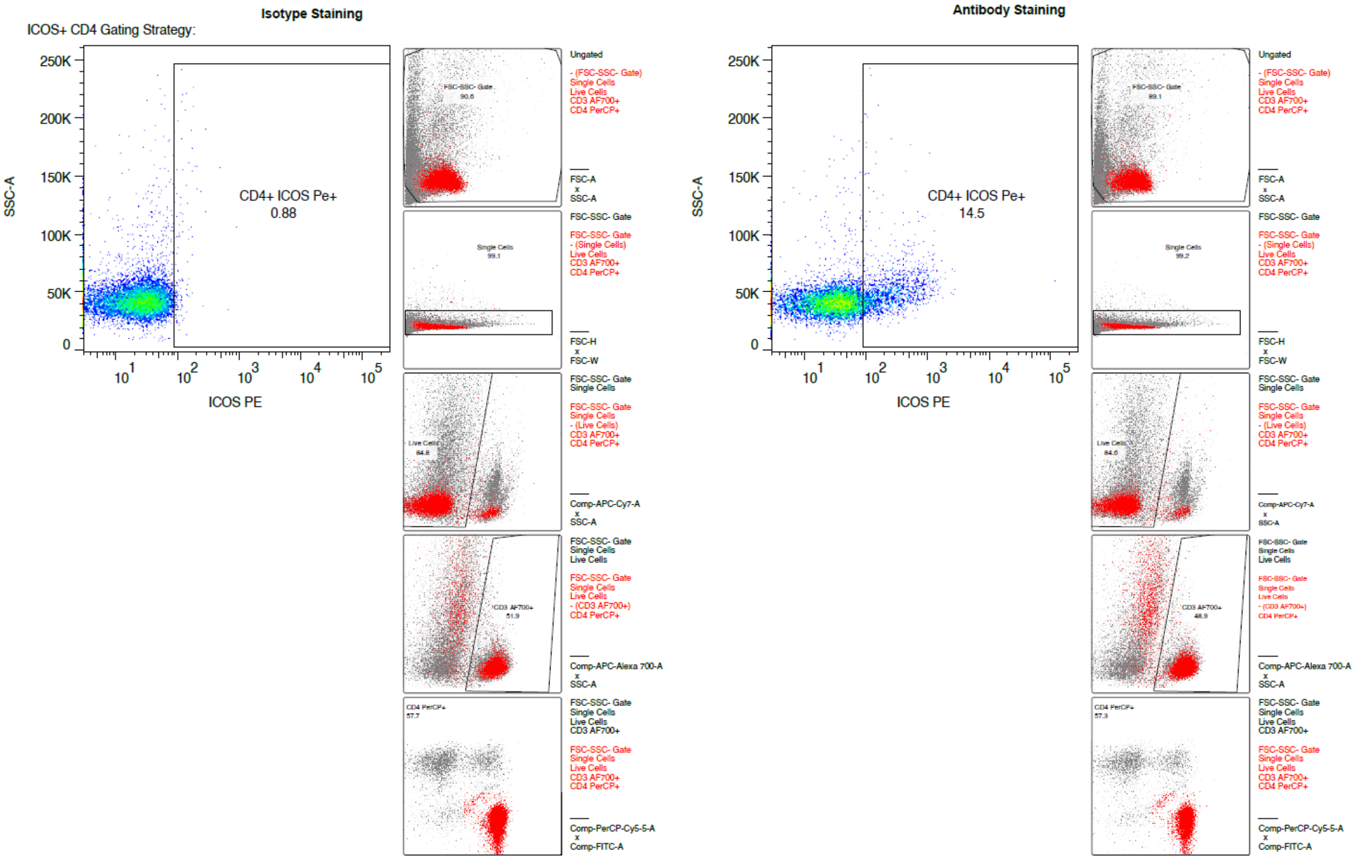

ICOS+ CD8 Gating Strategy:

Isotype Staining

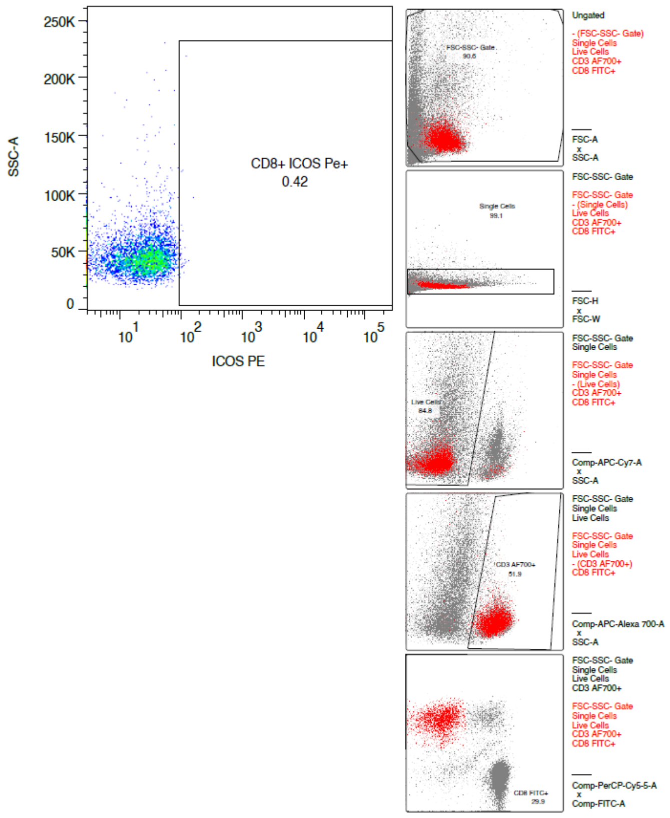

Antibody Staining

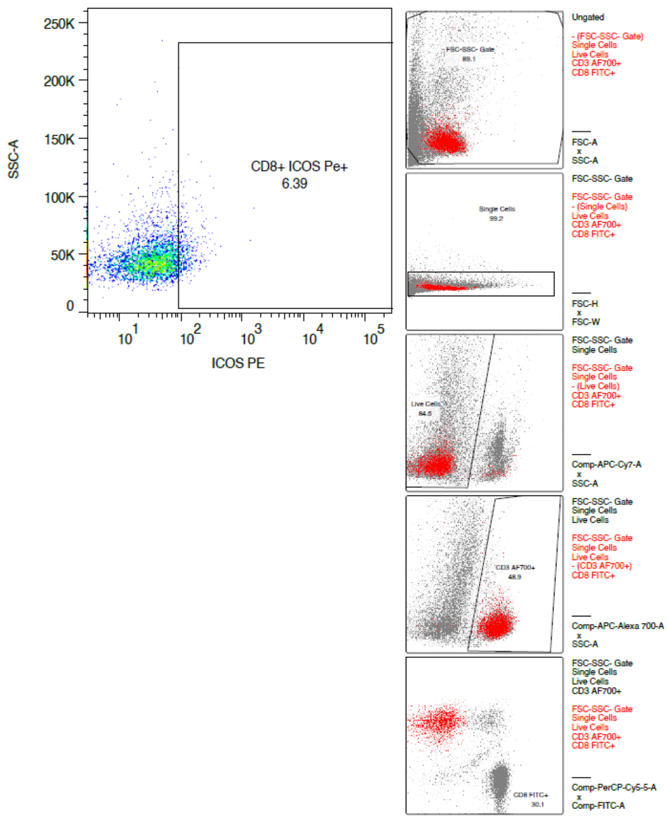

T Regulatory Cell Gating Strategy:

Isotype Staining

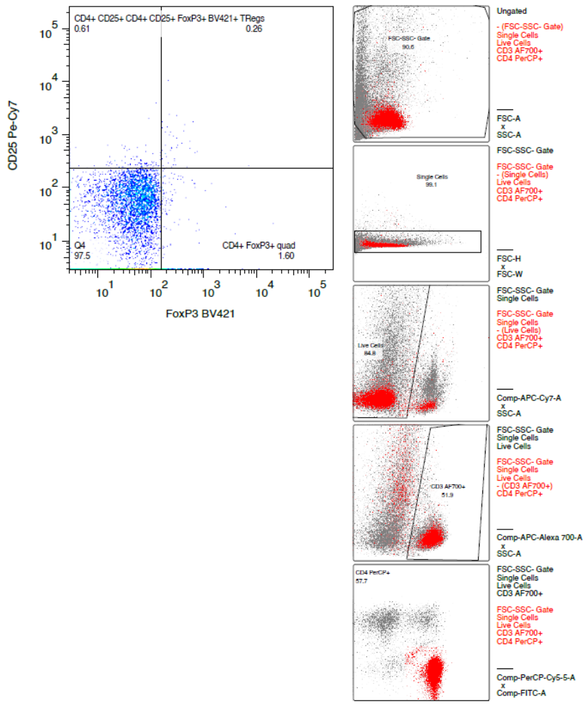

Antibody Staining

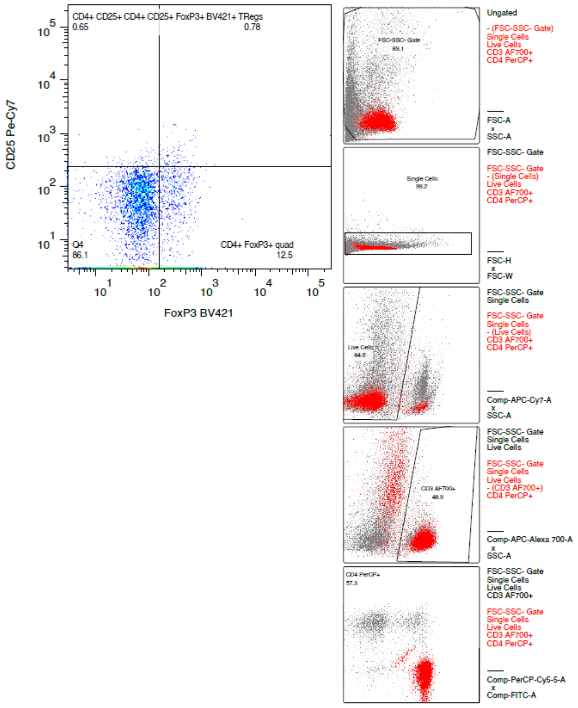

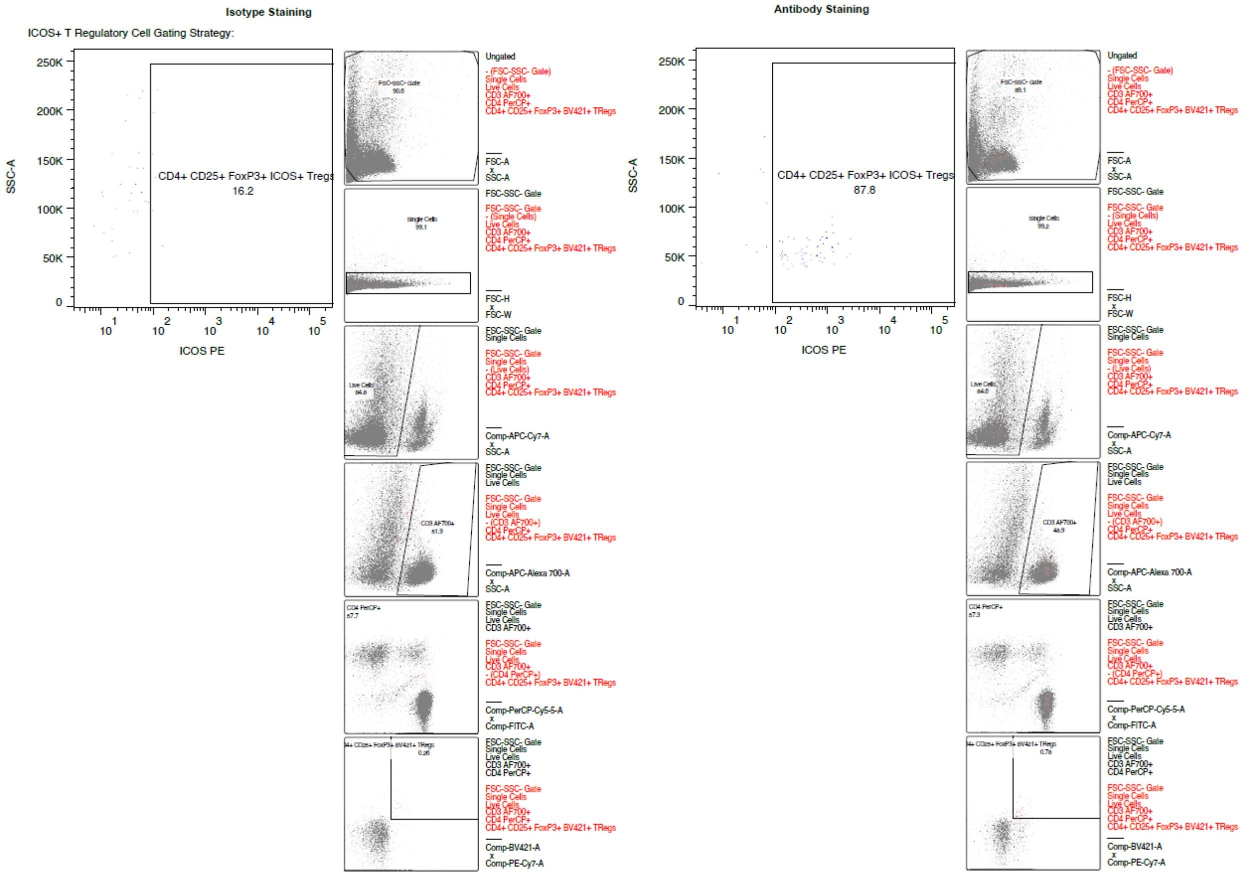

Supplementary Fig. 2. Flow cytometry gating strategy for ICOS on individual T cell populations. Associated with **Fig. 1d**, **e**.
