# Supplementary material for: Activating Inducible T-cell Costimulator Yields Antitumor Activity Alone and in Combination with Anti-PD-1 Checkpoint Blockade
Source: Cancer Res Commun. 2023 Aug 16;3(8):1564–79. doi: 10.1158/2767-9764.CRC-22-0293 (PMC10430783; doi:10.1158/2767-9764.CRC-22-0293)
Supplement: Supplementary Figure 3 — Affinity and kinetics [file crc-22-0293-s06.pdf]

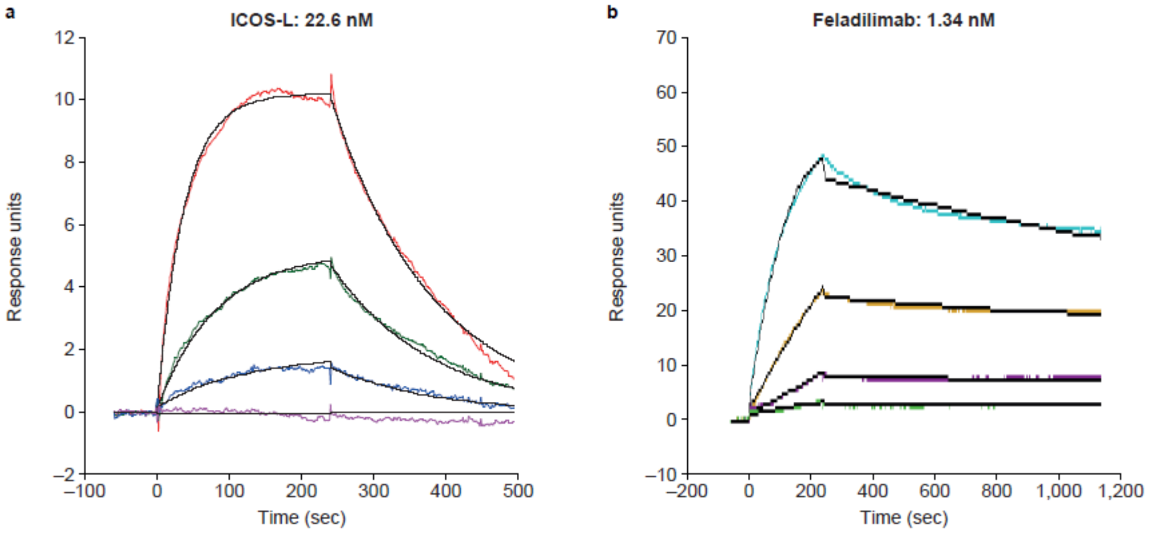

c

Sample	Target	ka	kd	KD (nM)
Human ICOS-L	Human ICOS-Fc	3.15E+05	7.13E–03	22.6
Feladilimab	Human ICOS-Fc	2.97E+05	3.96E–04	1.34

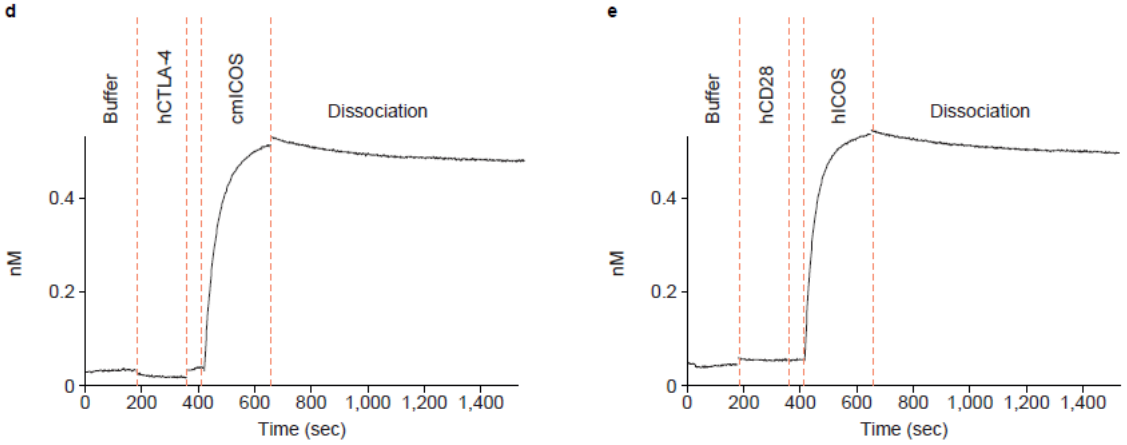

Supplementary Fig. 3. Affinity and kinetics of (**a**) recombinant human ICOS-L or (**b**) feladilimab binding to rabbit Fc-tagged recombinant human or cynomolgus monkey ICOS were determined using SPR. (**c**) Tabulation of the association (ka, $M^{-1}s^{-1}$), dissociation (kd, s^{-1}), and equilibrium (KD, nM) constants for feladilimab and ICOS-L binding to recombinant human ICOS (hICOS). (**d, e**) Binding kinetics of feladilimab (Fc variant: hIgG1) to (**d**) recombinant

human CTLA-4 (hCTLA-4) and (**e**) recombinant human CD28 (hCD28), as determined by Octet. In (**d**), cmICOS refers to recombinant cynomolgus monkey ICOS. Data in (**d, e**) are normalized to reference sensor (buffer only).
