# Supplementary material for: Activating Inducible T-cell Costimulator Yields Antitumor Activity Alone and in Combination with Anti-PD-1 Checkpoint Blockade
Source: Cancer Res Commun. 2023 Aug 16;3(8):1564–79. doi: 10.1158/2767-9764.CRC-22-0293 (PMC10430783; doi:10.1158/2767-9764.CRC-22-0293)
Supplement: Supplementary Figure 4 — Uncropped gel images [file crc-22-0293-s07.pdf]

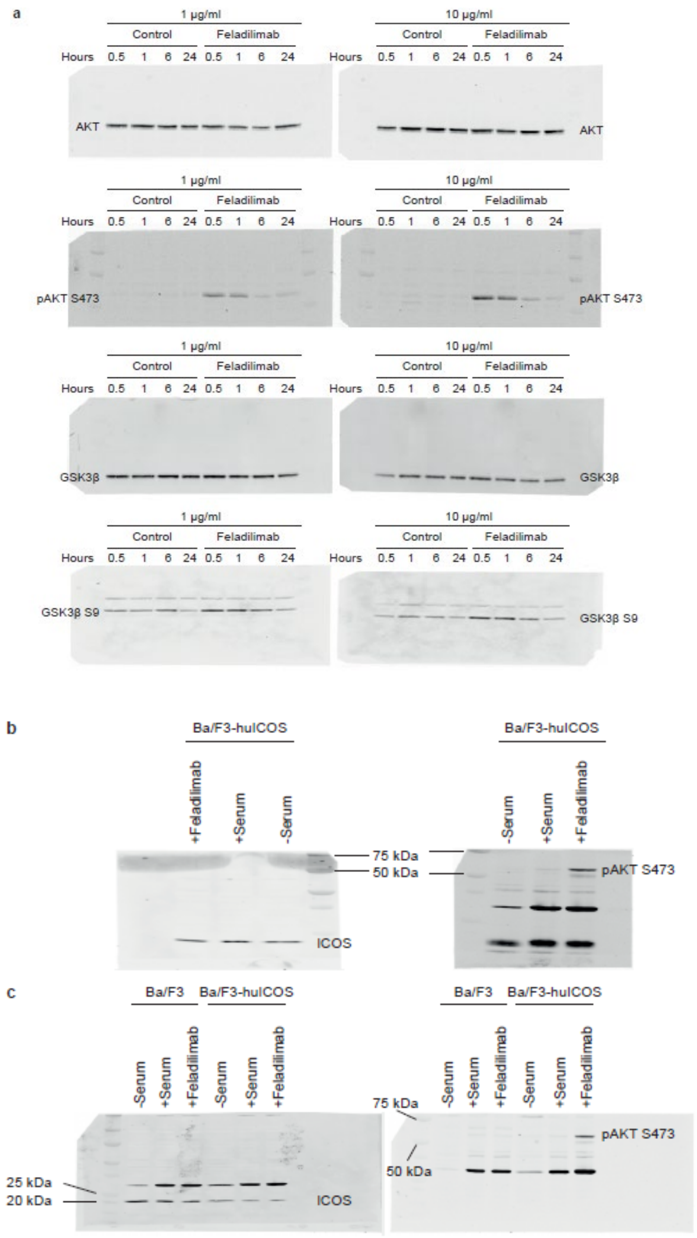

Supplementary Fig. 4. Uncropped gel images for (a) **Fig. 2d**, (b) **Supplementary Fig. 5a**, and (c) **Supplementary Fig. 5b**.
