# Supplementary material for: Activating Inducible T-cell Costimulator Yields Antitumor Activity Alone and in Combination with Anti-PD-1 Checkpoint Blockade
Source: Cancer Res Commun. 2023 Aug 16;3(8):1564–79. doi: 10.1158/2767-9764.CRC-22-0293 (PMC10430783; doi:10.1158/2767-9764.CRC-22-0293)
Supplement: Supplementary Figure 5 — Feladilimab induces phospho-AKT in ICOS-expressing Ba/F3 cells and cytokine production in PBMC from healthy donors and cancer patients. [file crc-22-0293-s08.pdf]

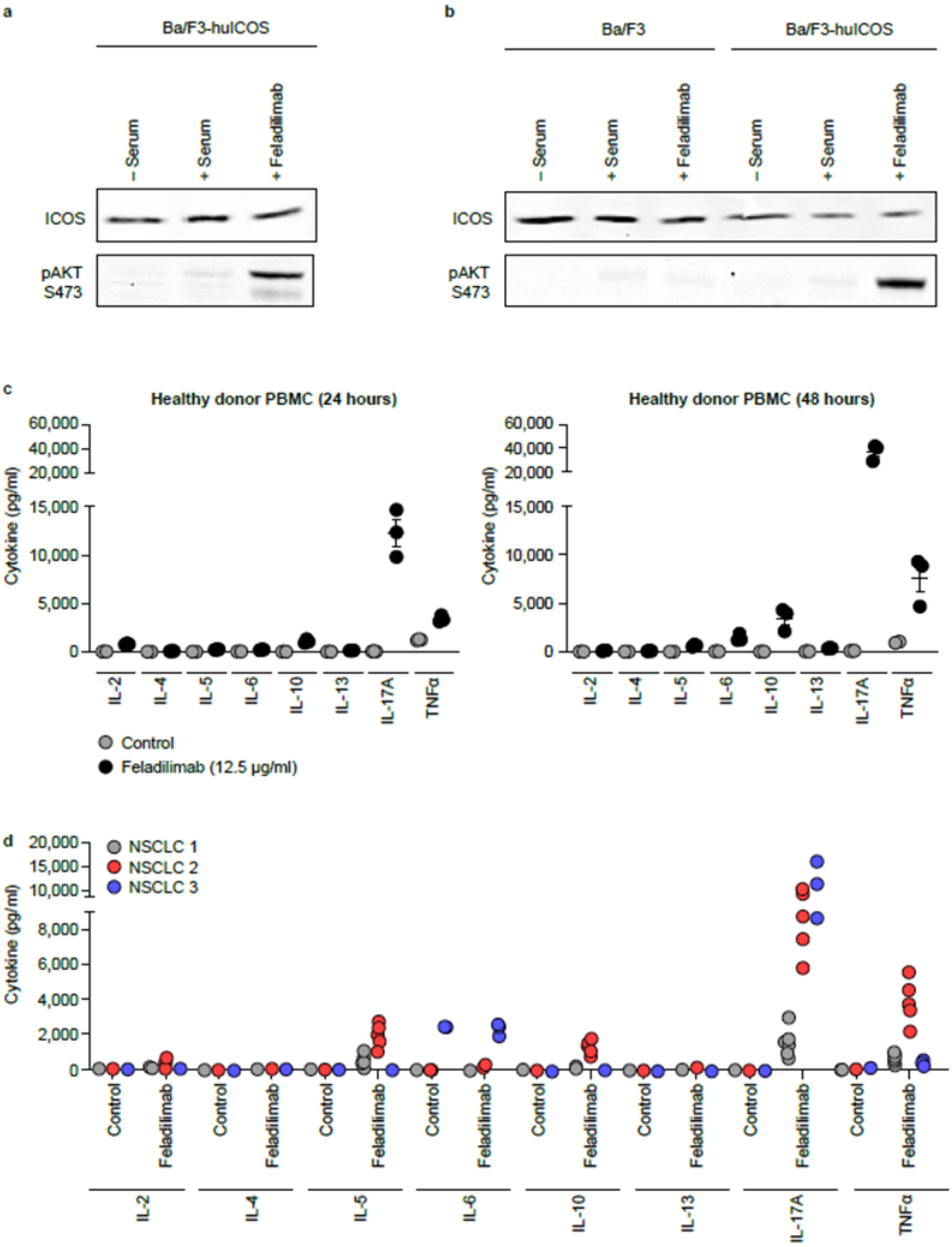

Supplementary Fig. 5. Feladilimab induces phospho-AKT in ICOS-expressing Ba/F3 cells and cytokine production in PBMC from healthy donors and cancer patients. (**a, b**) Ba/F3 (mouse) cells with or without expression of human ICOS (Ba/F3-huICOS) were treated with feladilimab (20 µg/ml) for 1 hour and assessed for changes in AKT phosphorylation. ICOS expression was readily detectable in both wild-type and Ba/F3-huICOS cells using a polyclonal human-mouse cross-reactive antibody (ab133680). (**a**) and (**b**) show data from two independent experiments; gels are cropped, uncropped gel images are available in **Supplementary Fig. 4 b**, **c**. (**c, d**) cytokine production in the supernatant of

PBMC cultures from (**c**) healthy donors and (**d**) NSCLC patients following stimulation with feladilimab and anti-CD3 (24 and 48 hours for healthy donors; 72 hours for NSCLC patients) (data representative of 3 independent experiments using PBMC from 3 different donors). Individual values are shown in (**c, d**), with bars representing the mean ± s.e.m.
