# Supplementary material for: Activating Inducible T-cell Costimulator Yields Antitumor Activity Alone and in Combination with Anti-PD-1 Checkpoint Blockade
Source: Cancer Res Commun. 2023 Aug 16;3(8):1564–79. doi: 10.1158/2767-9764.CRC-22-0293 (PMC10430783; doi:10.1158/2767-9764.CRC-22-0293)
Supplement: Supplementary Figure 6 — Feladilimab-mediated ICOS co-stimulation results in differential phenotypic changes and cytokine production in CD4+ non-Treg and Treg populations. [file crc-22-0293-s09.pdf]

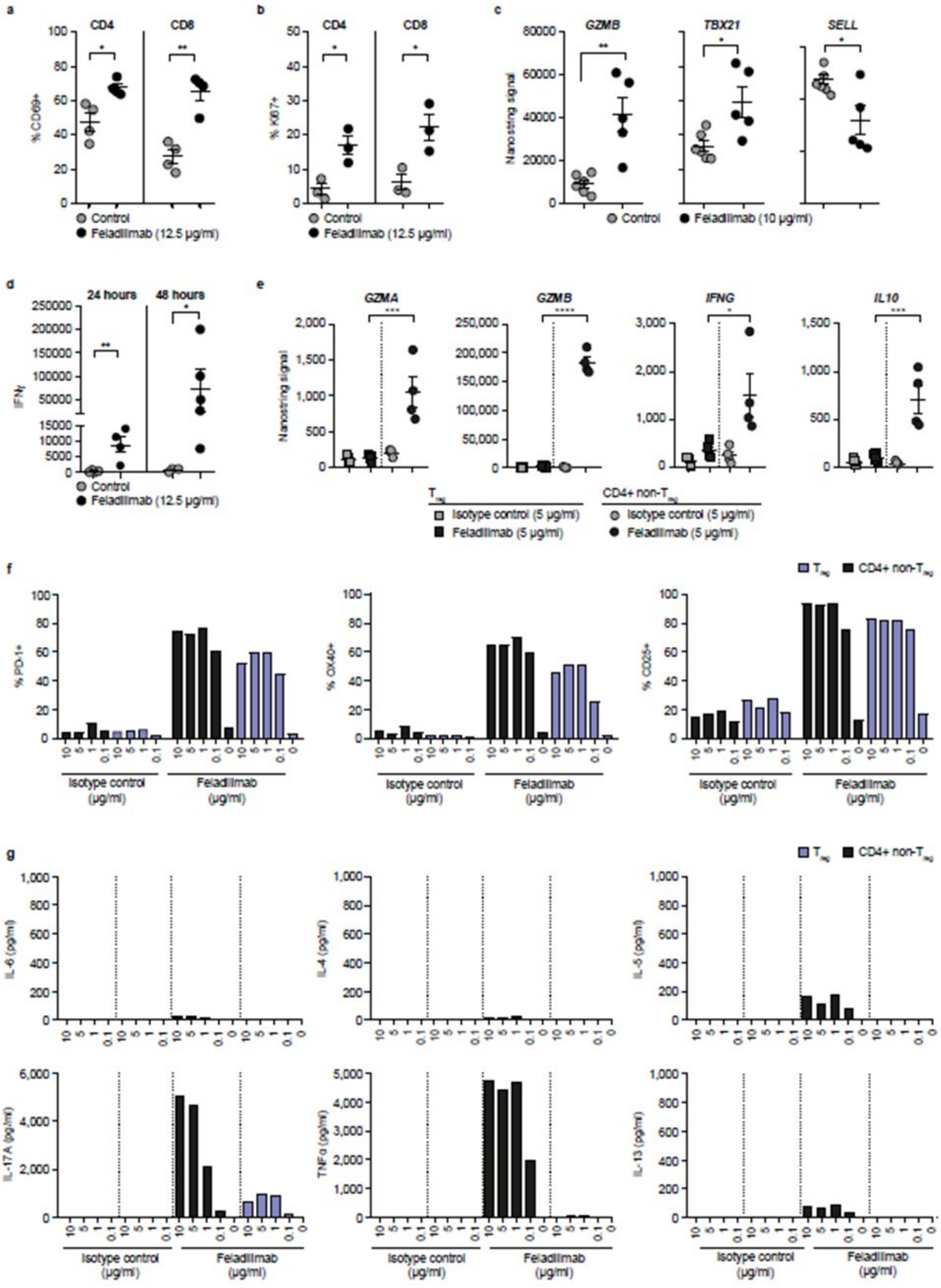

Supplementary Fig. 6. Feladilimab-mediated ICOS co-stimulation results in differential phenotypic changes and cytokine production in CD4+ non-T_{reg} and T_{reg} populations.

Flow cytometry-based expression of (**a**) CD69 and (**b**) Ki67 on healthy donor PBMCs following concurrent exposure to plate-bound anti-CD3 (0.6 µg/ml) and feladilimab or isotype control (12.5 µg/ml) for 48 hours. (**c**) RNA-based analysis of *GZMB*, *TBX21*, and *SELL* expression in purified CD3+ T cells following concurrent exposure to plate-bound anti-CD3 (0.6 µg/ml) and feladilimab or isotype control (10 µg/ml) for 24 hours. (**d**) IFNγ production in the supernatant of PBMC cultures from healthy donors following plate-bound feladilimab and anti-CD3 (0.6 µg/ml) stimulation (24 and 48 hours for healthy donors; 72 hours for NSCLC patients). (**e**) RNA-based analysis (Nanostring) of effector cell/functional marker (*GZMA*, *GZMB*, *TFNG*, *IL10*) expression by the stimulated cell subsets (each symbol represents an individual donor). (**f-g**) As illustrated in **Fig. 2e**, CD4+ non-T_{reg} (CD4+ CD25-) and T_{reg} cells (CD4+ CD25+ $CD127_{low}$) were isolated from healthy donor peripheral blood and stimulated using plate-bound anti-CD3 (1 µg/ml) ± feladilimab or isotype control (each at 5 µg/ml) for 72 hours. (**f**) Flow cytometry (nonspecific binding was blocked with human or mouse Fc block) and (**g**) cytokine-based analysis of T cell subsets following stimulation with plate-bound anti-CD3 and a dose range of feladilimab or isotype control; see **Supplementary Fig. 1** for gating strategy. Data in (**a–d**) represent the mean ± s.e.m; significance determined by unpaired Student's *t*-test.
