# Supplementary material for: Activating Inducible T-cell Costimulator Yields Antitumor Activity Alone and in Combination with Anti-PD-1 Checkpoint Blockade
Source: Cancer Res Commun. 2023 Aug 16;3(8):1564–79. doi: 10.1158/2767-9764.CRC-22-0293 (PMC10430783; doi:10.1158/2767-9764.CRC-22-0293)
Supplement: Supplementary Figure 7 — Healthy human donor CD4+ T cells were pre-activated with antiCD3/CD28 for 48 hours and then re-stimulated with plate-bound anti-CD3 in the presence of increasing concentrations of soluble or plate-bound feladilimab or isotype control for 72 hours. IFNg levels were then assessed from cell-free supernatants using Meso Scale Discovery (MSD)-based detection. [file crc-22-0293-s10.pdf]

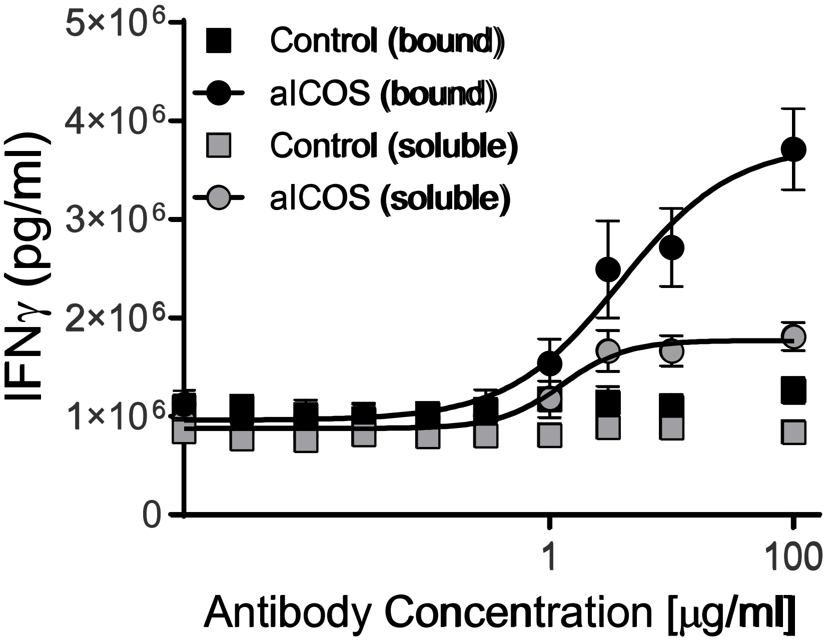

Supplementary Fig. 7. Healthy human donor CD4+ T cells were pre-activated with anti-CD3/CD28 for 48 hours and then re-stimulated with plate-bound anti-CD3 in the presence of increasing concentrations of soluble or plate-bound feladilimab or isotype control for 72 hours. IFNg levels were then assessed from cell-free supernatants using Meso Scale Discovery (MSD)-based detection. Error bars represent mean ± standard deviation; significance determined by unpaired student's *t*-test.
