# Supplementary material for: Activating Inducible T-cell Costimulator Yields Antitumor Activity Alone and in Combination with Anti-PD-1 Checkpoint Blockade
Source: Cancer Res Commun. 2023 Aug 16;3(8):1564–79. doi: 10.1158/2767-9764.CRC-22-0293 (PMC10430783; doi:10.1158/2767-9764.CRC-22-0293)
Supplement: Supplementary Figure 8 — Pharmacokinetic analysis of the mIgG1 anti-ICOS antibody (7E.17G9) in the peripheral blood of EMT6 tumor-bearing mice. [file crc-22-0293-s11.pdf]

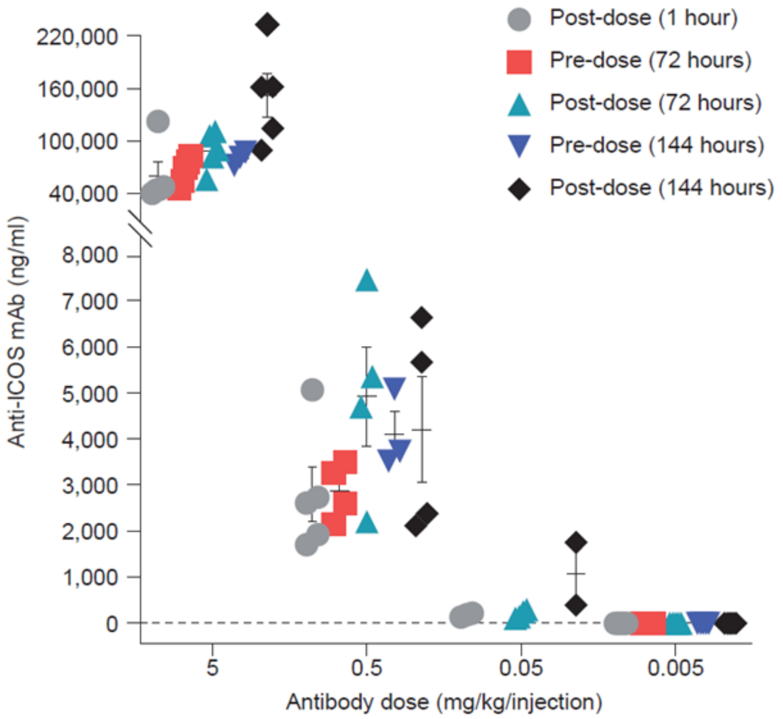

Supplementary Fig. 8. Pharmacokinetic analysis of the mIgG1 anti-ICOS antibody (7E.17G9) in the peripheral blood of EMT6 tumor-bearing mice. Serum samples were collected 1-hour post-dose and both pre-dose and 1-hour post-dose at 72 and 144 hours for characterization of antibody presence. Due to limitations in assay sensitivity, anti-ICOS antibody at the 0.05 mg/kg dose was not detectable in 72-hour and 144-hour pre-dose samples. No anti-ICOS antibody was detected in the 0.005 mg/kg dose cohort. Data are represented as mean ± s.e.m. of n=4–5 individual mice.
