# Supplementary material for: Activating Inducible T-cell Costimulator Yields Antitumor Activity Alone and in Combination with Anti-PD-1 Checkpoint Blockade
Source: Cancer Res Commun. 2023 Aug 16;3(8):1564–79. doi: 10.1158/2767-9764.CRC-22-0293 (PMC10430783; doi:10.1158/2767-9764.CRC-22-0293)
Supplement: Supplementary Figure 9 — Antitumor activity of feladilimab in a humanized patient-derived xenograft model of triple-negative breast cancer. [file crc-22-0293-s12.pdf]

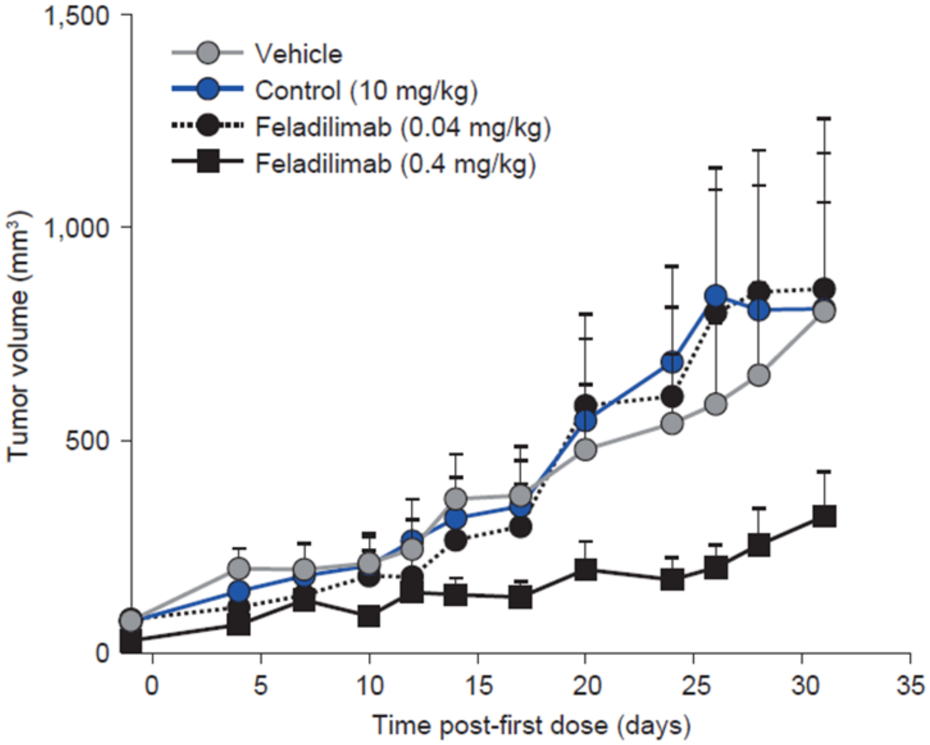

Supplementary Fig. 9. Antitumor activity of feladilimab in a humanized patient-derived xenograft model of triple-negative breast cancer. Following human CD34+ HPSC engraftment, female NSG mice (n=7–8/group) were implanted with breast cancer tumors (BR1126) and administered i.p. with feladilimab or control when tumors reached an average tumor volume of ~70 mm^3. Human CD34+ HPSC-engrafted mice were added to the study on a rolling basis. Each group represents the mean tumor volume (mm^3) ± s.e.m. at each evaluable time point.
