# Supplementary material for: Activating Inducible T-cell Costimulator Yields Antitumor Activity Alone and in Combination with Anti-PD-1 Checkpoint Blockade
Source: Cancer Res Commun. 2023 Aug 16;3(8):1564–79. doi: 10.1158/2767-9764.CRC-22-0293 (PMC10430783; doi:10.1158/2767-9764.CRC-22-0293)
Supplement: Supplementary Figure 10 — Rationale for combination of feladilimab with anti–PD-1 blockade. [file crc-22-0293-s13.pdf]

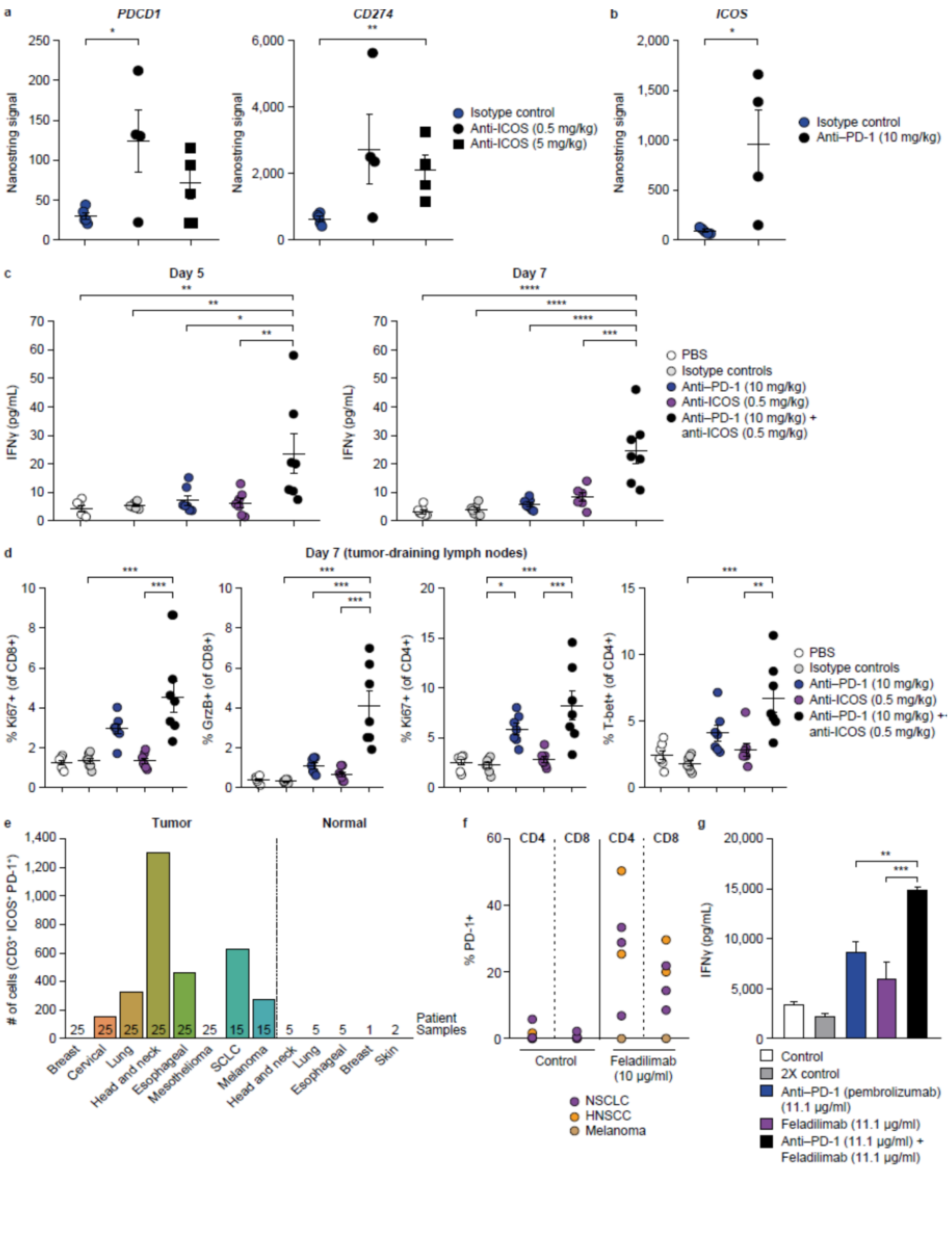

Supplementary Fig. 10. Rationale for combination of feladilimab with anti–PD-1 blockade. (**a**) RNA-based expression of PD-1 (*PDCD1*) and PD-L1 (*CD274*) on TILs isolated from anti-ICOS mAb (7E.17G9 rIgG2b)–treated EMT6 tumor-bearing mice on study day 7 (48 hours post final dose). (**b**) As in (**a**), *ICOS* expression following treatment with anti–PD-1 mAb (RMP1-14 rIgG2a). (**c**) IFNγ levels in the serum of EMT6 tumor-bearing

mice 48 hours post second (study day 5) and third doses (study day 7) of indicated mono- or combination therapies (7E.17G9 mIgG1 and RMP1-14 rIgG2a). (**d**) % Ki67+ CD8+ T cells, granzyme B (GrzB)+ CD8+ T cells, Ki67+ CD4+ T cells, and T-bet+ CD4+ T cells in the tumor-draining lymph nodes of EMT6 tumor-bearing mice 48 hours post third dose (study day 7) of indicated mono- or combination therapies (7E.17G9 mIgG1 and RMP1-14 rIgG2a) assessed using flow cytometry (nonspecific binding was blocked with human or mouse Fc block). (**e**) Quantification of the co-expression of CD3+ PD-1+ ICOS+ cells in both normal tissue and tumor biopsies obtained from different tumor types by high-order multiplex immunofluorescence (MultiOmyx platform, NeoGenomics). (**f**) Flow cytometry analysis of % PD-1+ T cell populations with and without plate-bound anti-CD3 antibody (0.6 μg/ml) and feladilimab (10 μg/ml) treatment for 72 hours (nonspecific binding was blocked with human or mouse Fc block); see **Supplementary Fig. 1** for gating strategy. (**g**) IFNγ production in a modified allogeneic MLR following exposure to feladilimab (11.1 μg/ml) and anti–PD-1 (pembrolizumab, 11.1 μg/ml) alone or in combination for 96 hours. 2X control represents the combination of two hIgG4-PE isotype controls. Error bars in (**a–d, g**) represent mean ± s.e.m. Significance was determined by unpaired *t*-test (two-tailed) in (**a, b**) and one-way ANOVA in (**c, d, g**).
