# Supplementary material for: Activating Inducible T-cell Costimulator Yields Antitumor Activity Alone and in Combination with Anti-PD-1 Checkpoint Blockade
Source: Cancer Res Commun. 2023 Aug 16;3(8):1564–79. doi: 10.1158/2767-9764.CRC-22-0293 (PMC10430783; doi:10.1158/2767-9764.CRC-22-0293)
Supplement: Supplementary Figure 11 — Clinical case study of feladilimab monotherapy. [file crc-22-0293-s14.pdf]

a

	Monotherapy case study
Disease/stage	• Stage IIIc nodular melanoma<br>• BRAF/c-KIT mutation negative
Prior treatment	• Ipilimumab/nivolumab ~ 2 months<br>• Nivolumab ~1 year
Study treatment	Feladilimab 1 mg/kg and 3 mg/kg for 48 weeks
Response	Partial response followed by stable disease

b

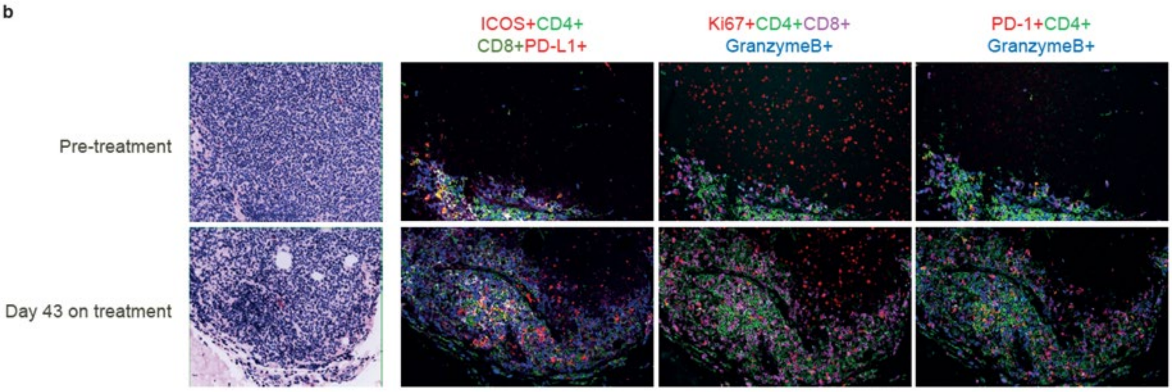

Supplementary Fig. 11. Clinical case study of feladilimab monotherapy. (**a**) Clinical history, treatment details, and response for a patient with melanoma who received feladilimab monotherapy and (**b**) corresponding tumor pharmacodynamic data. See **Supplementary Fig. 12a** for CT imaging of tumor lesions for this patient.
