# Supplementary material for: Activating Inducible T-cell Costimulator Yields Antitumor Activity Alone and in Combination with Anti-PD-1 Checkpoint Blockade
Source: Cancer Res Commun. 2023 Aug 16;3(8):1564–79. doi: 10.1158/2767-9764.CRC-22-0293 (PMC10430783; doi:10.1158/2767-9764.CRC-22-0293)
Supplement: Supplementary Figure 12 — Clinical case studies of feladilimab monotherapy and in combination with pembrolizumab. [file crc-22-0293-s15.pdf]

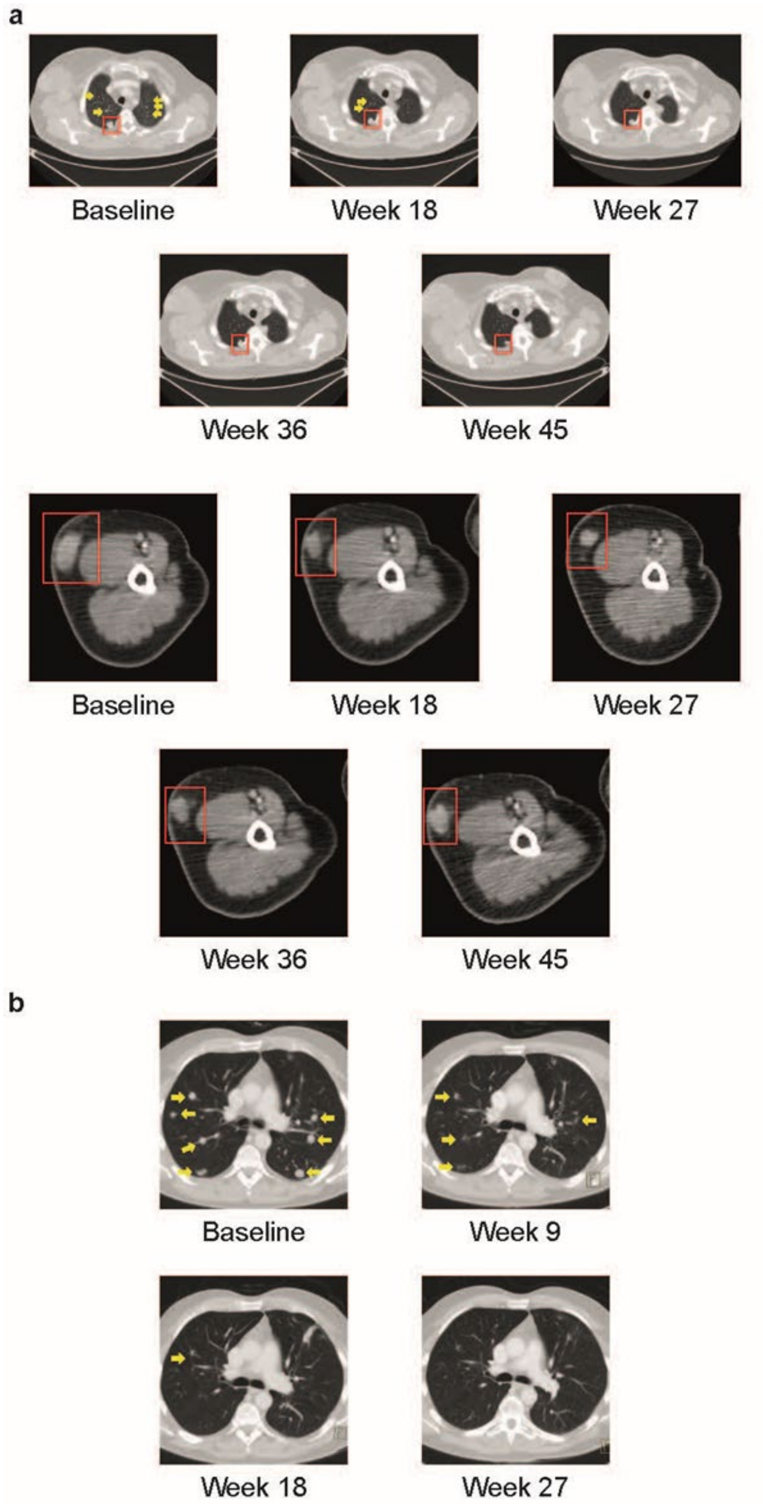

Supplementary Fig. 12. Clinical case studies of feladilimab monotherapy and in combination with pembrolizumab. (**a**) CT imaging of tumor lesions for a patient with metastatic nodular melanoma who received feladilimab monotherapy at 1 mg/kg followed by 3 mg/kg to week 48 (patient presented in **Supplementary Fig 11a, b**). (**b**) CT imaging of

tumor lesions for the patient with oropharyngeal squamous cell carcinoma who received feladilimab at 0.3 mg/kg + pembrolizumab 200 mg Q3W to week 30 (patient presented in **Fig. 5a**, **c**).
