# Supplementary material for: Activating Inducible T-cell Costimulator Yields Antitumor Activity Alone and in Combination with Anti-PD-1 Checkpoint Blockade
Source: Cancer Res Commun. 2023 Aug 16;3(8):1564–79. doi: 10.1158/2767-9764.CRC-22-0293 (PMC10430783; doi:10.1158/2767-9764.CRC-22-0293)
Supplement: Supplementary Table 1 — Antibodies used for in vitro and in vivo assays, and experimental model [file crc-22-0293-s16.pdf]

Supplementary Table 1. Antibodies used for *in vitro* and *in vivo* assays, and experimental models

Assay	Antibody	Clone	Manufacturer	RRID
Flow cytometry	CD4 (anti-human)	RPA-T4	BD Biosciences	BD Biosciences Cat# 560468, RRID:AB_1645271
	CD4 (anti-mouse)	RM4-5	BD Biosciences	BD Biosciences Cat# 550954, RRID:AB_393977
	CD8 (anti-human)	RPA-T8	BioLegend	BioLegend Cat# 301032, RRID:AB_893422
	CD8 (anti-mouse)	53-6.7	BD Biosciences	BD Biosciences Cat# 560182, RRID:AB_1645237
	CD69 (anti-human)	FN50	BioLegend	BioLegend Cat# 310911, RRID:AB_314846
	Ki67 (anti-human)	B56	BD Biosciences	BD Biosciences Cat# 556026, RRID:AB_396302
	Ki67 (anti-mouse)	16A8	BioLegend	BioLegend Cat# 652420, RRID:AB_2564285
	ICOS (anti-human)	ISA3	eBioscience	Thermo Fisher Scientific Cat# 12-9948-42, RRID:AB_10732348

	ICOS (anti-mouse)	C398.4a	BioLegend	BioLegend Cat# 313524, RRID:AB_2562545
	FOXP3 (anti-human)	PCH101	eBioscience	Thermo Fisher Scientific Cat# 12-4776-42, RRID:AB_1518782
	FOXP3 (anti-mouse)	Fjk-16s	eBioscience	Thermo Fisher Scientific Cat# 17-5773-82, RRID:AB_469457
	PD-1 (anti-human)	EH12.2H7	BioLegend	BioLegend Cat# 329908, RRID:AB_940475
	PD-1 (anti-mouse)	RMP1-30	BioLegend	BioLegend Cat# 109110, RRID:AB_57201
	CD25 (anti-human)	BC96	BiolegendBioLegend	BioLegend Cat# 302632, RRID:AB_11218989
	CD25 (anti-mouse)	PC61	BD Biosciences	BD Biosciences Cat# 552880, RRID:AB_394509
	CD45 (anti-human)	H130	BD Biosciences	BD Biosciences Cat# 560566, RRID:AB_1645452
	CD45 (anti-mouse)	30-F11	BD Biosciences	BD Biosciences Cat# 563053, RRID:AB_2737976

	CD3 (anti-human)	UCHT1	BioLegend	BioLegend Cat# 300424, RRID:AB_493741
	CD3 (anti-mouse)	145-2C11	BD Biosciences	BD Biosciences Cat# 553062, RRID:AB_394595
	T-bet (anti-mouse)	O4-46	BD Biosciences	BD Biosciences Cat# 561268, RRID:AB_10564071
	granzyme B (anti-mouse)	GB11	BioLegend	BioLegend Cat# 515405, RRID:AB_2294995
	CD127 (anti-mouse)	A7R34	BioLegend	BioLegend Cat# 135012, RRID:AB_1937216
	CD44 (anti-mouse)	IM7	BioLegend	BioLegend Cat# 103039, RRID:AB_10895752
	OX40 (anti-human)	ACT35	eBioscience	Thermo Fisher Scientific Cat# 11-1347-42, RRID:AB_10597448
Western blot	pAKT (S473 and Thr308)	D9E	Cell Signaling Technology	Cell Signaling Technology Cat# 4060, RRID:AB_2315049
	Total AKT	9272	Cell Signaling Technology	Cell Signaling Technology Cat#

				9272, RRID:AB_329827
	pGSK3β (Ser9)	D85E12	Cell Signaling Technology	Cell Signaling Technology Cat# 5558, RRID:AB_10013750
	Total GSK3β	D5C5Z	Cell Signaling Technology	Cell Signaling Technology Cat# 12456, RRID:AB_2636978
	ICOS (human-mouse cross-reactive)	ab133680 (polyclonal Ab)	Abcam	Discontinued, not in system
Immunofluorescence	ICOS	SP98	Abcam	Abcam Cat# ab105227, RRID:AB_10710236
	PD-1	EPR4877(2)	Abcam	Abcam Cat# ab137132, RRID:AB_2894867
	PD-L1	SP142	Abcam	Abcam Cat# ab228462, RRID:AB_2827816
	OX40/CD134	Ber-ACT35	BioLegend	BioLegend Cat# 350002, RRID:AB_10639951
	CD16	DJ130c	ThermoFisher	Thermo Fisher Scientific Cat# MA1-84008, RRID:AB_2104009

	CD56	MRQ-42	Cell Marque	Nolan lab - Stanford Cat# 156R, RRID:AB_2864402
	Ki67	SP6	SpringBio	Spring Bioscience Cat# M3061, RRID:AB_1661312
	HLA-DR	WR18	Novus	Novus Cat# NB100-64358, RRID:AB_964563
	granzyme B	GrB-7	Dako	Agilent Cat# M7235, RRID:AB_2114697
	CD8	C8/144B	Dako	Agilent Cat# M7103, RRID:AB_2075537
	CD3	F7.2.38	Dako	Agilent Cat# M7254, RRID:AB_2631163
	CD4	EPR6855	Abcam	Abcam Cat# ab133616, RRID:AB_2750883
	S100	Polyclonal Ab	Dako	Agilent Cat# GA50461-2, RRID:AB_2811056
	PanCytokeratin	PCK-26	Sigma-Aldrich	Sigma-Aldrich Cat# C5992, RRID:AB_2134432

Experimental models	**Cell line**	**Species**	**Source**	**RRID**
	EMT6	Mouse	ATCC	ATCC Cat# CRL-2755, RRID:CVCL_1923
	A549	Human	ATCC	RRID:CVCL_0023
	A2058	Human	ATCC	RRID:CVCL_1059
	BR1126 (TNBC PDX)	Human	The Jackson Laboratory	None, patient-derived
	BA/F3	Mouse	DSMZ	DSMZ Cat# ACC-300, RRID:CVCL_0161
	**Mice**	**Use**	**Source**	**RRID**
	BALB/c	Syngeneic	Harlan/Envigo	RRID:IMSR_APB:4790
	NOD SCID gamma (NSG)	Humanized	The Jackson Laboratory	RRID:IMSR_JAX:005557
In vivo treatment	**Antibody**	**Clone**	**Manufacturer**	**RRID**
	ICOS	7E.17G9 rIgG2b	BioXcell	Bio X Cell Cat# BE0059, RRID:AB_1107622
	ICOS	7E.17G9 mIgG1	Absolute Antibody	Custom
	PD-1	RMP1-14 rIgG2a	BioXcell	Bio X Cell Cat# BE0146, RRID:AB_10949053
	rIgG2b isotype control	LTF2	BioXcell	Bio X Cell Cat# BE0090, RRID:AB_1107780
	mIgG1 isotype control	MOPC21	BioXcell	Bio X Cell Cat# BE0083, RRID:AB_1107784

	rIgG2a isotype control	2A3	BioXcell	Bio X Cell Cat# BE0089, RRID:AB_1107769
