# Supplementary material for: Activating Inducible T-cell Costimulator Yields Antitumor Activity Alone and in Combination with Anti-PD-1 Checkpoint Blockade
Source: Cancer Res Commun. 2023 Aug 16;3(8):1564–79. doi: 10.1158/2767-9764.CRC-22-0293 (PMC10430783; doi:10.1158/2767-9764.CRC-22-0293)
Supplement: Supplementary Table 5 — Pearson correlation (r) analysis of ICOS versus PD-L1 (CD274) in different tumor type. [file crc-22-0293-s20.pdf]

Supplementary Table 5. Pearson correlation (r) analysis of *ICOS* versus PD-L1 (*CD274*) in different tumor types

**Indication**	**r**	***P* value**
**ICOS vs CD274 r value ranking**		
BRCA	0.6248	0
BLCA	0.4408	0
LUAD	0.3931	0
SKCM	0.3931	4.01E-05
PAAD	0.3661	5.03E-07
HNSC	0.3611	0
ESCA	0.2878	7.43E-05
CESC	0.2638	3.11E-06
SARC	0.2516	4.20E-05
LUSC	0.1864	2.68E-05
**ICOS vs ICOSLG r value ranking**		
PAAD	0.3455	2.32E-06
LUSC	0.3185	2.85E-13
HNSC	0.2901	1.53E-11
CESC	0.2323	4.31E-05
SKCM	0.2157	0.028685259
BRCA	0.1708	1.07E-08
LUAD	0.1698	8.78E-05
ESCA	0.1549	0.035793078
BLCA	0.1271	0.009925008
SARC	0.1137	0.067779107
**ICOSLG vs CD274 r value ranking**		
LUAD	0.0869	0.045958733
BRCA	0.0826	0.005991044
CESC	0.05	0.385013142
SKCM	0.045	0.651732174
LUSC	0.0316	0.480256577
HNSC	0.0098	0.82325339
PAAD	-0.0076	0.919604834
BLCA	-0.0205	0.679178622
SARC	-0.0512	0.411526463
ESCA	-0.1333	0.071278803

Key:

**Correlation**	**r range**
Strong positive	0.5-1
Moderate positive	0.3-0.49
Low positive	0.1-0.29
Minimal	0-0.09

BLCA, bladder cancer (n=411); BRCA, breast cancer (n=1107); CESC, cervical cancer (n=304); ESCA, esophageal cancer (n=184); HNSC, head and neck squamous cell carcinoma (n=520); LUAD, lung adenocarcinoma (n=528); LUSC, lung squamous cell carcinoma (n=501); PAAD, pancreatic cancer (n=178); SARC, sarcoma (n=259); SKCM, melanoma (n=103). Values in parentheses represent the number of patient specimens per indication.
