# Supplementary material for: Activating Inducible T-cell Costimulator Yields Antitumor Activity Alone and in Combination with Anti-PD-1 Checkpoint Blockade
Source: Cancer Res Commun. 2023 Aug 16;3(8):1564–79. doi: 10.1158/2767-9764.CRC-22-0293 (PMC10430783; doi:10.1158/2767-9764.CRC-22-0293)
Supplement: Supplementary Table 6 — Example TIL marker expression phenotypes from clinical patient case studies of feladilimab monotherapy and in combination with pembrolizumab. [file crc-22-0293-s21.pdf]

Supplementary Table 6. Example TIL marker expression phenotypes from clinical patient case studies of feladilimab monotherapy and in combination with pembrolizumab

	Fig 5b (monotherapy)		Fig 5c (combination with pembrolizumab)		Supplementary Fig 11 (monotherapy)	
**Phenotype (cells/mm^2)**	**Pre-treatment**	**On-treatment**	**Pre-treatment**	**On-treatment**	**Pre-treatment**	**On-treatment**
CD3+	19	241	320	2435	224	2669
CD3+CD4+	14	148	80	642	71	908
CD3+CD8+	0	72	167	1520	137	1449
CD16+CD56+	0	0	36	52	5	33
CD3+CD8+ GRANZYMEB+	0	23	44	421	61	470
PD1+	0	23	146	1074	111	1240
PDL1+	0	62	8042	1108	170	1020
CD3+CD8+HLADR+	0	26	44	1018	36	433
Ki67+	715	833	2707	280	2260	1701
CD3+CD8+Ki67+	0	10	29	80	36	215
CD3+CD4+Ki67+	0	12	7	25	9	84
CD3+CD4+FOXP3+ Ki67+	0	6	7	5	5	39
CD3+CD4+CD134+	0	23	22	79	21	310
CD3+CD8+CD134+	0	2	15	51	8	180
